# New bioactive secondary metabolites from fungi: 2023

**DOI:** 10.1080/21501203.2024.2354302

**Published:** 2024-06-11

**Authors:** Ying Shi, Minhui Ji, Jiayu Dong, Dongxiao Shi, Yitong Wang, Longhui Liu, Shuangshuang Feng, Ling Liu

**Affiliations:** a State Key Laboratory of Mycology, Institute of Microbiology, Chinese Academy of Sciences, Beijing, China; b University of Chinese Academy of Sciences, Beijing, China

**Keywords:** Fungal natural products, novel structures, chemical investigations, annual summary, pharmaceutical effects, research strategies

## Abstract

Fungi have been identified as a prolific source of structurally unique secondary metabolites, many of which display promising biological and pharmacological properties. This review provides an overview of the structures of new natural products derived from fungi and their biological activities along with the research strategies, which focuses on literature published in the representative journals in 2023. In this review, a total of 553 natural products including 219 polyketides, 145 terpenoids, 35 steroids, 106 alkaloids, and 48 peptides are presented. By summarising the latest findings, this review aims to provide a guide and inspire further innovation in the fields of the discovery of fungal natural products and pharmaceutical development.

## GRAPHICAL ABSTRACT

**Figure:**
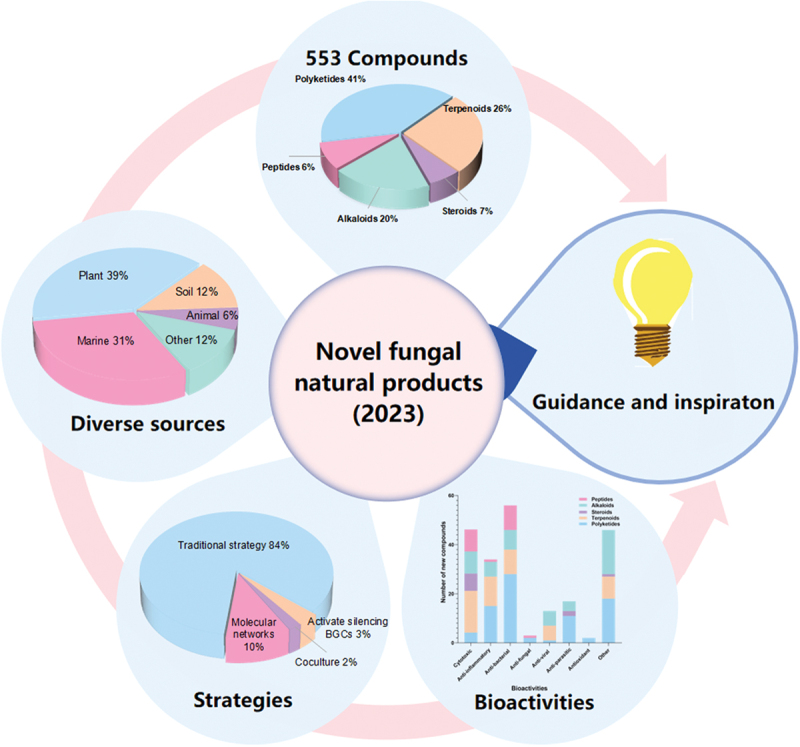

## 1. Introduction

Secondary metabolites obtained from natural resources have gained considerable attention due to their potential as key components in new drug development (Evidente 2022). Natural products are usually isolated from various sources such as plants, animals, marine organisms, fungi, bacteria, and others (Hui et al. 2023). They exhibit a wide range of pharmacophores and a high degree of stereochemistry, which are expected to contribute to their strong pharmacological properties (Tammam et al. 2023). Furthermore, with the development and progress of research technology, the investigations on drugs based on natural products are entering a new era (Schor and Cox 2018; Saldívar et al. 2022). Fungi are distributed widespread in nature and have been recognised as one of the important sources of natural products due to their abundant secondary metabolites biosynthetic gene clusters (BGCs) (Hautbergue et al. 2018; Yee et al. 2023). Many fungal-derived natural products possess unique structures and display diverse biological properties (Lin et al. 2021; Holland and Carroll 2023). Since the discovery of penicillin by Alexander Fleming in 1928, many fungal natural products and derivatives have been used as drugs (Molnár et al. 2010), such as lipid-lowering medications (lovastatin), immunosuppressants (cyclosporine and mycophenolic acid), and vasoconstrictors (ergometrine) (Orfali et al. 2021). Therefore, the investigation of fungal secondary metabolites plays an important role in drug development.

Nevertheless, fungal genome research on the identified species suggests that over 80% of their secondary metabolites remain unknown (Simpson 2014), indicating that a large number of compounds are still waiting to be discovered (Rateb and Ebel 2011). Therefore, it is significant to accumulate the chemical structures, bioactivities, and research strategies of the new natural products reported recently. This allows us to summarise research paradigms and propose new breakthrough points in combination with interdisciplinary intersections. Consequently, employing the key words “fungi natural products”, “fungi secondary metabolites”, “fungal chemistry”, and “fungi mycotoxin”, this review insights into the literature published in 2023 by searching on PubMed, Web of Science, ACS, RSC, Springer Link, Elsevier, and Wiley databases. Finally, we focus on the representative journals in the field of natural products, including the *Journal of the American Chemical Society*, *Angewandte Chemie International Edition*, *Chemical Science*, *Organic Letters*, *Bioorganic Chemistry*, *Journal of Natural Products*, *Journal of Agriculture and Food Chemistry*, *Chemical Communications*, *Chinese Chemical Letters*, *Planta Medica*, *Journal of Organic Chemistry*, and *Chinese Journal of Natural Medicine*, etc. This review identifies 553 compounds, including 219 polyketides, 145 terpenoids, 35 steroids, 106 alkaloids, and 48 peptides, isolated from the endophytic fungi, marine-derived fungi, solid-associated fungi, animal-derived fungi, and other origin fungi. In this review, we systematically summarise and analyse the structures and biological activities of the new compounds, fungal strain sources and research strategies, aiming to offer new insights into the field of fungal natural products.

## 2. Source and strategy of fungi for chemical studies

**Figure 1.**
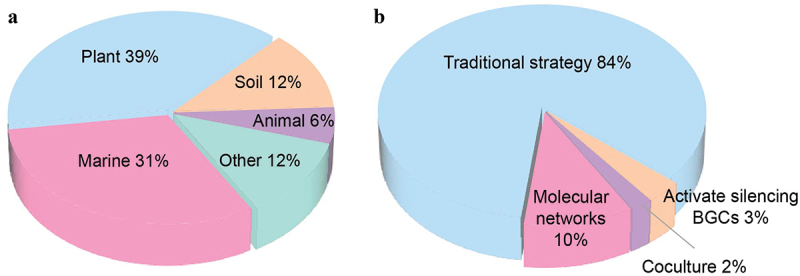
Number of new compounds derived from fungi in 2023. (a) Divided by sources of fungal strains. (b) Divided by research strategies.

Figure 1a provides an overview of new compounds isolated from fungi in 2023, categorised by their sources of fungal strains. Based on the literature research results, the fungal sources of all the new compounds reported in 2023 can be divided into four parts, including plants (endophytic fungi and phytopathogenic fungi are both involved), soil, marine, and animals. According to the data from Figure 1, it can be estimated that over one-third of the new compounds reported during 2023 are derived from plants, with an approximately even split between plant and marine sources in the broadest terms, while the remaining compounds originate from fungi isolated from soil, animals and other sources.

Screening bioactive secondary metabolites from such a vast fungal resource for drug research is a challenging task (Newman and Cragg 2020). It is also difficult to elucidate the structure of unknown fungal secondary metabolites due to their complexity and low amounts. Fortunately, advances in high-throughput untargeted metabolomics have led to the development of bioinformatics and cheminformatics tools. Some of the promising strategies for natural product research were conducted in the recent study (Figure 1b) including molecular networking, NMR-guided separation, co-culture techniques, and activation of silent BGCs [One Strain Many Compounds (OSMAC) strategy and heterologous expression]. However, the largest proportion of new compounds were discovered using traditional strategies (including bioactivity-guided isolation), exceeding 80%. Based on the statistical results from Figure 1b, molecular networking is the most widely used strategy during natural products research, accounting for approximately 10% of the number of new compounds. Scientists prefer traditional strategies such as bio-guided fraction purification to investigate the secondary metabolites of the fungi, with the direct aim of exploring new bioactive natural products and contributing to drug development. Interestingly, almost 70% literatures reviewed herein have proposed the possible biosynthetic pathways of the isolated compounds, highlighting the significance of analysing biosynthetic pathways and expanding databases of BGCs for natural product research. In this review, the biosynthetic pathways of representative compounds for every structural type (except for steroids due to the lack of relevant reports) are summarised.

The overview information of the new compounds including their fungal sources, and strain names, together with the bioactivities and study strategies are summarised and organised in Table 1. With the bioactive compounds numbers attached in parentheses of the line “bioactivities”, it is estimated that less than half of the 553 compounds exhibited biological effects, including cytotoxic, anti-inflammatory, anti-bacterial, anti-viral, anti-fungal, anti-parasitic, anti-oxidant, and other activities (e.g. organ protection, plant growth regulation, and anti-Alzheimer’s, etc).

**Table 1.** Fungal sources of new natural products mentioned in this review along with their bioactivities and study strategies sources.

Fungal sources	Fungal strains	Compound No.	Bioactivities	Strategies	Reference
Plant	*Trichocladium crispatum*	**1**−**8**	Improving osteoporosis (**7**)	MN	Han et al. (2023)
Soil	*Talaromyces adpressus*	**9**−**15**	Anti-inflammatory (**13**, **14**)	T	Zheng et al. (2023)
Marine	*Stagonospora* sp. SYSU-MS7888	**16**−**24**	Anti-inflammatory (**16**, **17**, **23**, **24**)	T	Wu et al. (2023)
Marine	*Spiromastix* sp. SCSIO F190	**25**−**28**	Anti-bacterial (**25**−**28)**	T	Cai et al. (2023)
Plant	*Lepteutypa* sp. KT4162	**29**−**33**	Anti-virual (**30**)	T	Miura et al. (2023)
Plant	*Subplenodomus* sp. CPCC 401465	**34**−**43**	Anti-bacterial (**34**, **38**, and **40)**	MN	Cai et al. (2023)
——	*Trichoderma afroharzianum* T-22	**44**−**48**	NA	AB	Yan et al. (2023)
Plant	*Clonostachys rosea*	**49**−**56**	Anti-bacterial (**49**, **50**, and **52)**	T	Yang et al. (2023)
Plant	*Daldinia pyrenaica* 047188	**57**−**60**	Antimelanogenic (**57**)	T	Lee et al. (2023)
Soil	*Phomopsis* sp. DHS-11	**61**−**64**	Cytotoxic (**61**, **63**, **64**)	T	Guo et al. (2023)
Soil	*Xylaria* sp. KYJ-15F	**65**, **66**	Anti-bacterial (**65**, **66**)	OSMAC	Gan et al. (2023)
Marine	*Aspergillus versicolor* PS108-62	**67**, **68**	NA	OSMAC	Magot et al. (2023)
Soil	*Xenoacremonium sinensis* ML-31	**69**−**72**	NA	AB	Liu et al. (2023)
Plant	*Phomopsis prunorum*	**73**−**79**	Pro-angiogenic (**75**, **76**, and **79**)	NG	Dai et al. (2023)
——	*Griseofulvania griseomyces*	**80**−**85**	Anti-inflammatory (**80** and **83**)	T	Liang et al. (2023)
Plant	*Phaeosphaeria* sp. SQ-510	**86**−**91**	*Anti-Arabidopsis thaliana* (**86**, **87**, **90**, and **91**)	T	Zhai et al. (2023)
Plant	*Ophiobolus cirsii* LZU-1509	**92**−**107**	Antioxident (**106**)	T	Guo et al. (2023)
Marine	*Penicillium steckii* SCSIO 41040	**108**−**115**	Anti-inflammatory (**109**)	T	Song et al. (2023)
Plant	*Paraphaeosphaeria* sp. KT4192	**116**−**119**	Cytotoxic (**118**, **119**)	T	Kanehara et al. (2023)
Nest	*Talaromyces* sp. CMB-MW102	**120**−**125**	NA	MN	Samarasekera et al. (2023)
——	*Calcarisporium arbuscula*	**126**−**129**	Cytotoxic (**128**)	AB	Dong et al. (2023)
Marine	*Aspergillus terreus* HT5	**130**	NA	T	Wang et al. (2023)
Plant	*Ustilago maydis* MZA96986	**131**−**136**	Anti-inflammatory (**133** and **135**)	T	Wu et al. (2023)
Plant	*Daldinia childae* 047219	**137**−**142**	NA	MN	Kim et al. (2023)
Plant	*Stryphnodendron adstringens*	**143**−**144**	Anti-bacterial (**143**)	T	Iantas et al. (2023)
Soil	*Lasiodiplodia pseudotheobromae* 414-JZ-40	**145**, **146**	Anti-inflammatory (**145** and **146**)	T	Liang et al. (2023)
Marine	*Peroneutypa* sp. M16	**147**−**153**	Anti-parasitic (**151)**	T	Oliveira et al. (2023)
Plant	*Rhexocercosporidium* sp. Dzf14	**154**−**163**	Anti-bacterial (**154**, **158**, **160**−**163**)	T	Gu et al. (2023)
Plant	*Pseudogymnoascus* sp. OUCMDZ-3578	**164**−**170**	Anti-Alzheimer (**169** and **170**)	T	Yin et al. (2023)
Plant	*Trichoderma koningiopsis*	**171**−**182**	Anti-inflammatory (**171** and **172**)	T	Huang et al. (2023)
Soil	*Aternaria* sp.	**183**−**186**	Cytotoxic (**185**)	T	Fu et al. (2023a)
Plant	*Resupinatus* sp. BCC84615	**187**, **188**	Anti-bacterial and cytotoxic (**187**, **188**)	T	Harms et al. (2023)
Marine	*Aspergillus* sp. CSIO41315	**189**–**209**	Neuraminidase inhibit (**189**)	T	Wei et al. (2023)
Soil	*Scytalidium* sp. IQ-074	**210**−**213**	*h*PTP1B_1–400_ inhibit (**211**)	T	Martinez et al. (2023)
Soil	*Trichocladium* sp.	**214**−**219**	NA	T	Lee et al. (2023)
Plant	*Psathyrella candolleana*	**220**−**226**	Cytotoxic (**220**)	T	Zhao et al. (2023)
Soil	*Phoma* sp. CGMCC 10481	**227**−**229**	Cytotoxic (**227**)	T	Li et al. (2023)
Plant	*Aspergillus nidulans*	**230**−**235**	Cytotoxic (**230**, **232**, **233**)	T	Fu et al. (2023b)
Plant	*Trichoderma citrinoviride* HT-9	**236**−**238**	Cytotoxic (**236**)	T	Yin et al. (2023)
Plant	*Antrodiella zonata*	**239**−**250**	Anti-bacterial (**239**, **243**, and **250**)	T	Gao et al. (2023)
Plant	*Xylaria hypoxylon*	**252**−**257**	Anti-bacterial (**251**, **253**, **254**, and **256**)	T	Miral et al. (2023)
Marine	*Aspergillus versicolor* YPH93	**258**−**264**	Cytotoxic (**264**)	T	Zheng et al. (2023)
Plant	*Bipolaris eleusines*	**265**−**274**	Phytotoxic (**266**−**269** and **272**−**273**); cytotoxic (**265**−**272**)	MN	Wei et al. (2023)
Marine	*Paraconiothyrium sporulosum*	**275**−**281**	Anti-inflammatory (**275**−**280**)	T	Sun et al. (2023)
Animal	*Aspergillus parasiticus* SDU001	**282**−**287**	Anti-inflammatory (**283** and **286**)	OSMAC	Dai et al. (2023)
Marine	*Amphichorda felina* SYSU-MS7908	**288**−**292**	Anti-inflammatory (**287**)	T	Jiang et al. (2023b)
Soil	*Scytalidium* sp. IQ-074	**293**, **294**	NA	T	Martinez et al. (2023)
——	*Aspergillus nomius* MST-FP2004 *Penicillium brasilianum* MST-FP1927	**295**	Anti-parasitic (**295**)	Coculture	Cowled et al. (2023)
——		**296**, **297**			
Plant	*Chaetomium globosum*	**298**, **299**	Anti-bacterial (**298**)	T	Morehouse et al. (2023)
Plant	*Bipolaris maydis*	**300**−**306**	Anti-inflammatory (**300**)	T	Shi et al. (2023)
Marine	*Paraconiothyrium hawaiiense* FS482	**307**–**311**	PAF-induced platelet aggregation (**307** and **311**)	T	Chen et al. (2023)
Soil	*Heimiomyces* sp. MUCL 56078	**312**−**317**	Cytotoxic (**317**)	T	Pfutze et al. (2023)
Plant	*Alternaria alternata* MB-30	**318**−**320**	Inhibitory effects on lipid accumulation (**318**)	T	Li et al. (2023)
Plant	*Abundisporus violaceus* MUCL 56355	**321**−**328**	Anti-bacterial (**321** and **323**)	T	Sum et al. (2023)
Soil	*Onygenales* sp. YX1425	**329**−**334**	Anti-parasitic (**330**)	T	Chen et al. (2023)
Plant	*Diaporthe* sp. XC1211	**335**−**346**	Anti-inflammatory (**342** and **346**)	T	Chang et al. (2023)
Marine	*Alternaria* sp. ZH-15	**347**, **348**	Antiepileptic (**347** and **348**)	T	Wang et al. (2023)
Marine	*Amphichorda felina* SYSU-MS7908	**349**−**352**	Cytotoxic (**351**)	T	Jiang et al. (2023c)
Soil	*Penicillium* sp. sb62	**353**−**357**	Anti-viral (**353**−**357**)	T	Chang et al. (2023)
——	*Aspergillus* sp. NF2396	**358**−**364**	Anti-bacterial (**360**, **361**, and **362**)	T	Salman et al. (2023)
Plant	*Ganoderma weberianum*	**365**−**386**	Antimalarial (**371** and **373**)	T	Chinthanom et al. (2023)
Marine	*Talaromyces adpressus*	**387**−**395**	Cytotoxic (**388**−**392** and **395**) Immunosuppressive (**393**)	T	Zheng et al. (2023)
Soil	*Aspergillus spectabilis*	**396**−**399**	Cytotoxic (**397**)	T	Wei et al. (2023)
Marine	*Penicillium janthinellium*	**400**–**408**	Anti-viral (**400**, **403**, **404**, and **406**–**408**)	Coculture	Cao et al. (2023)
Animal	*Paecilomyces formosus*	**409**–**419**	Heart transplant agents (**410** and **413**)	T	Jin et al. (2023)
	*Aspergillus clavatonanicus*				
Marine	*Aspergillus clavutus* LZD32-24	**420**–**440**	NA	T	Guo et al. (2023)
——	*Emericella* sp. 1454	**441**, **442**	Cytotoxic (**442**)	T	Chen et al. (2023)
Plant	*Boeremia exigua*	**443**–**446**	Cytotoxic (**443**–**446**)	T	Shi et al. (2023)
Plant	*Chaetomium nigricolor* F5	**447**–**451**	Cell relaxin (**447**)	T	Gu et al. (2023)
Plant	*Aspergillus amoneus* TJ507	**452**	Liver protective (**452**)	T	Zhang et al. (2023)
Marine	*Penicillium oxalicum* QDU1	**453**−**463**	Anti-inflammatory (**453**, **456**, **457**, **460**, **461**, and **463**)	T	Wu et al. (2023)
Plant	*Aspergillus* sp. GZWMJZ-258	**464**	Cytotoxic (**464**)	T	Wang et al. (2023)
Plant	*Sarocladium* sp. MSX6737	**465**–**467**	Cytotoxic (**467**)	T	Al Subeh et al. (2023)
Soil	*Fuligo septica*	**468**, **469**	NA	T	Minns et al. (2023)
Marine	*Biatriospora* sp. CBMAI 1333	**470**	(PAF) receptor antagonist activity (**470**)	T	Oliveira et al. (2023)
Plant	*Phaeosphaeria* sp.	**471**–**476**	Plant growth regulatory (**471**–**476**)	T	Zhai et al. (2023)
Plant	*Colletotrichum gloeosporioides* NRRL 45420	**477**–**480**	Plant growth inhibit (**477**–**480**)	T	Zhou et al. (2023)
Marine	*Aspergillus alabamensi*	**481**–**488**	Anti-bacterial (**481**–**488**)	T	Hu et al. (2023)
Plant	*Penicillium* sp. DG23	**489**−**496**	HMG-CoA reductase (**489**)	T	Su et al. (2023)
——	*Nigrospora* sp.	**497**−**501**	Anti-malarial parasitic (**497**−**501**)	MN	Yang et al. (2023)
Marine	*Exophiala mesophila* MCCC 3A00939	**502**−**505**	Cytotoxic (**502** and **503**)	T	Cheng et al. (2023)
Marine	*Aspergillus pseudoviridinutans* TW585	**506**−**512**	Anti-inflammatory (**511**)	MN	Ding et al. (2023)
Marine	*Tolypocladium* sp.	**513**, **514**	Anti-bacterial (**513** and **514**)	T	Morehouse et al. (2023)
Marine	*Beauveria felina*	**515**−**520**	Anti-fungal (**516** and **517**)	T	Jiang et al. (2023a)
Marine	*Aspergillus* insuetus SD-512	**521**−**525**	Anti-bacterial (**524** and **525**)	T	Chi et al. (2023)
Soil	*Sesquicillium* sp. q0466	**526**−**529**	Anti-bacterial (**526**−**529**)	T	Xiao et al. (2023)
Plant	*Elsinoe* sp.	**530**	Anti-fungal (**530**)	T	Du et al. (2023)
Soil	*Basidiobolus meristosporus* Drechsler	**531**−**533**	Cytotoxic (**531**, **532**)	T	Zhao et al. (2023)
Marine	*Trichoderma* sp. GXIMD 01001	**534**−**540**	Cytotoxic (**534**−**540**)	T	Lin et al. (2023)
Soil	*Trichoderma* sp.	**541**−**553**	Anti-bacterial (**541**−**547**)	T	Cheng et al. (2023)

“——” represents no source for the fungal strain was provided, “NA” for no activities were detected, “MN” represents molecular network, “T” for traditional strategy, and “AB” for active silencing BGCs.

## 3. Chemistry and biological activities

### 3.1. General aspects of secondary metabolites

**Figure 2.**
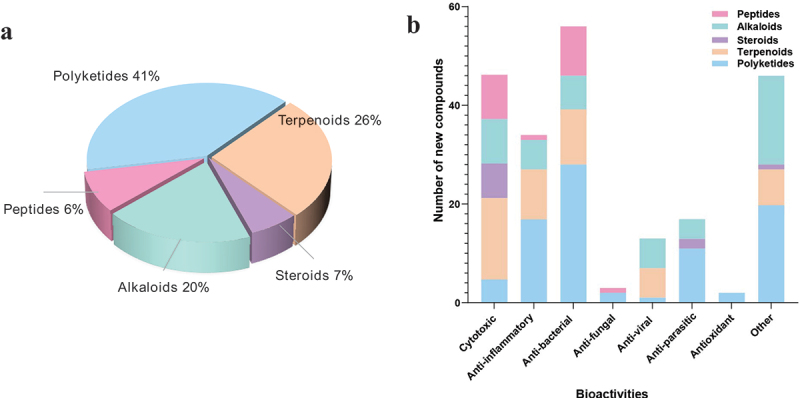
Number of new compounds derived from fungi in 2023. (a) Divided by structural types. (b) Separated by both bioactivities and structural conditions.

**Figure 3.**
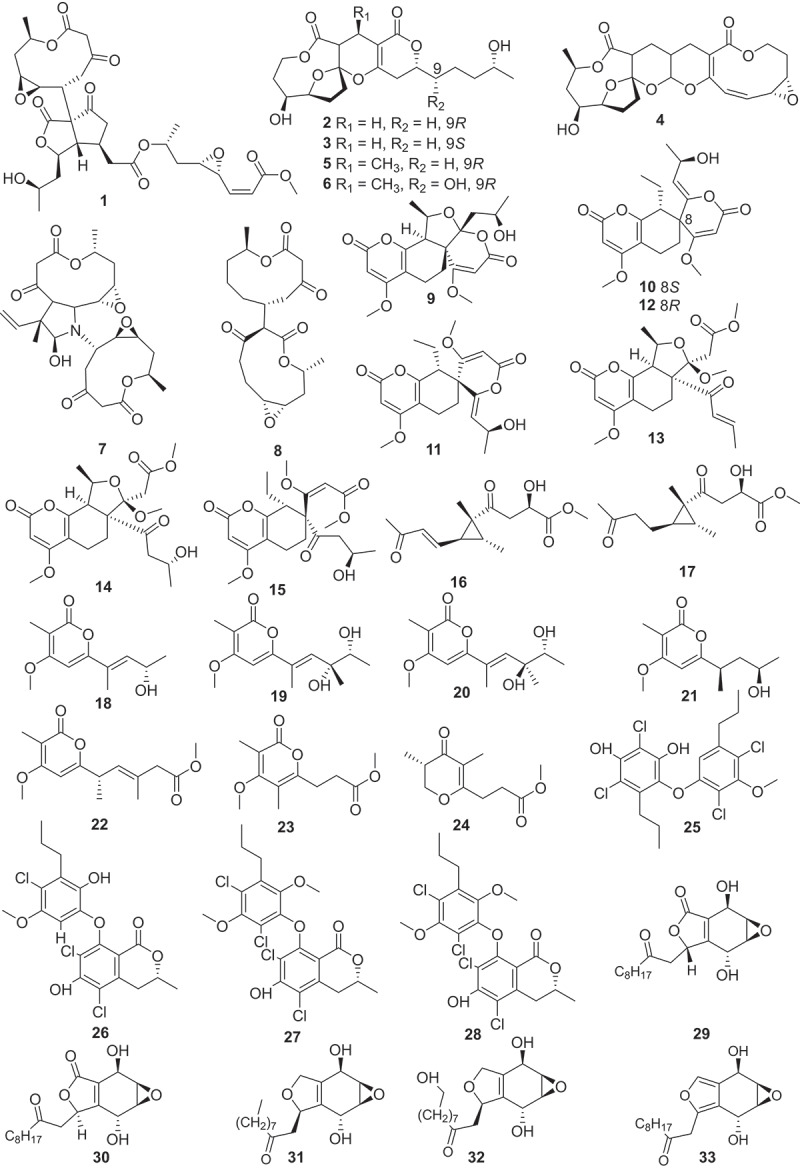
Structures of compounds **1**−**33**.

**Figure 4.**
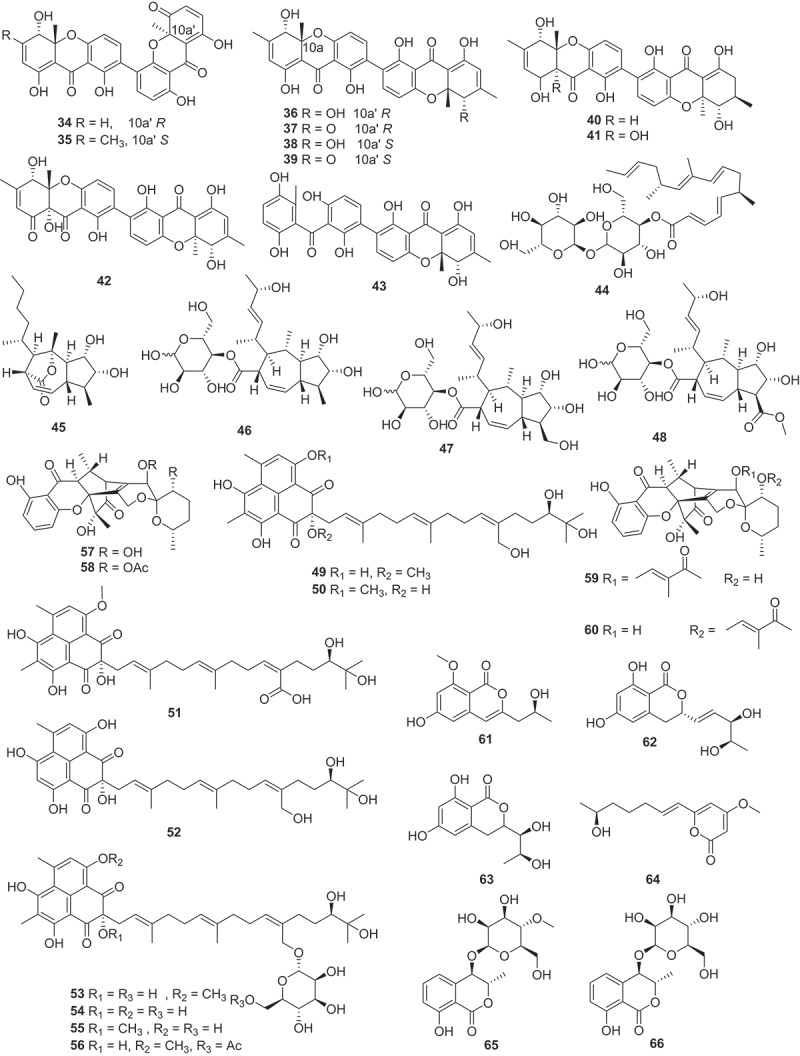
Structures of compounds **34**−**66**.

**Figure 5.**
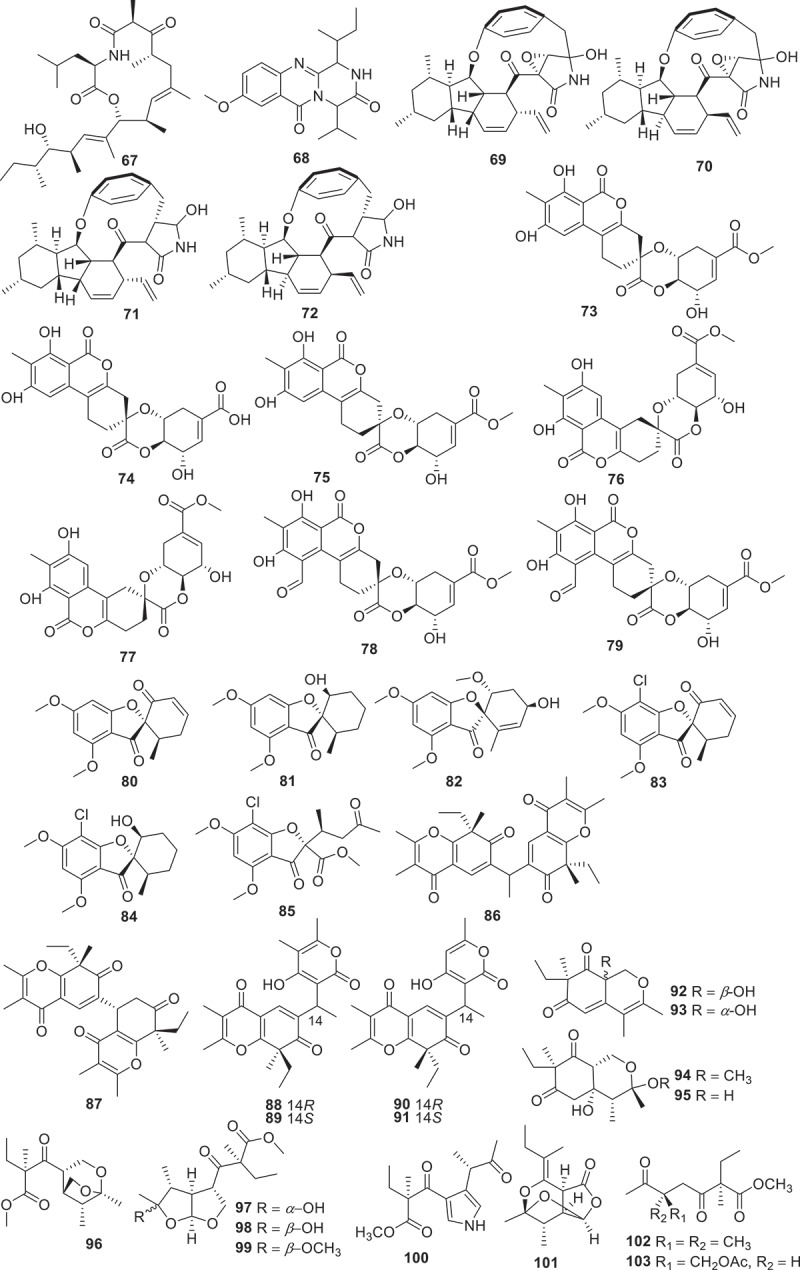
Structures of compounds **67**−**103**.

**Figure 6.**
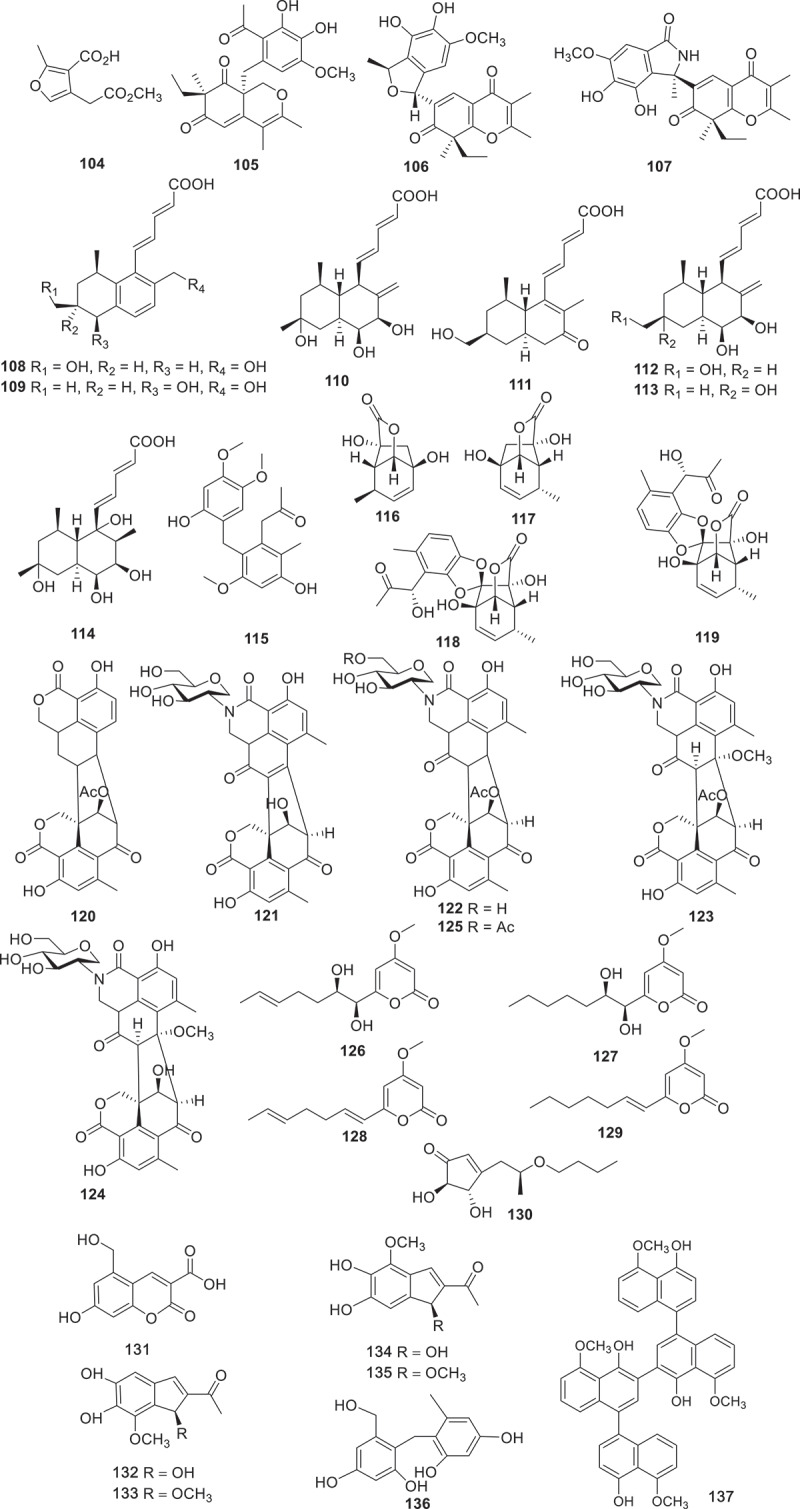
Structures of compounds **104**−**137**.

**Figure 7.**
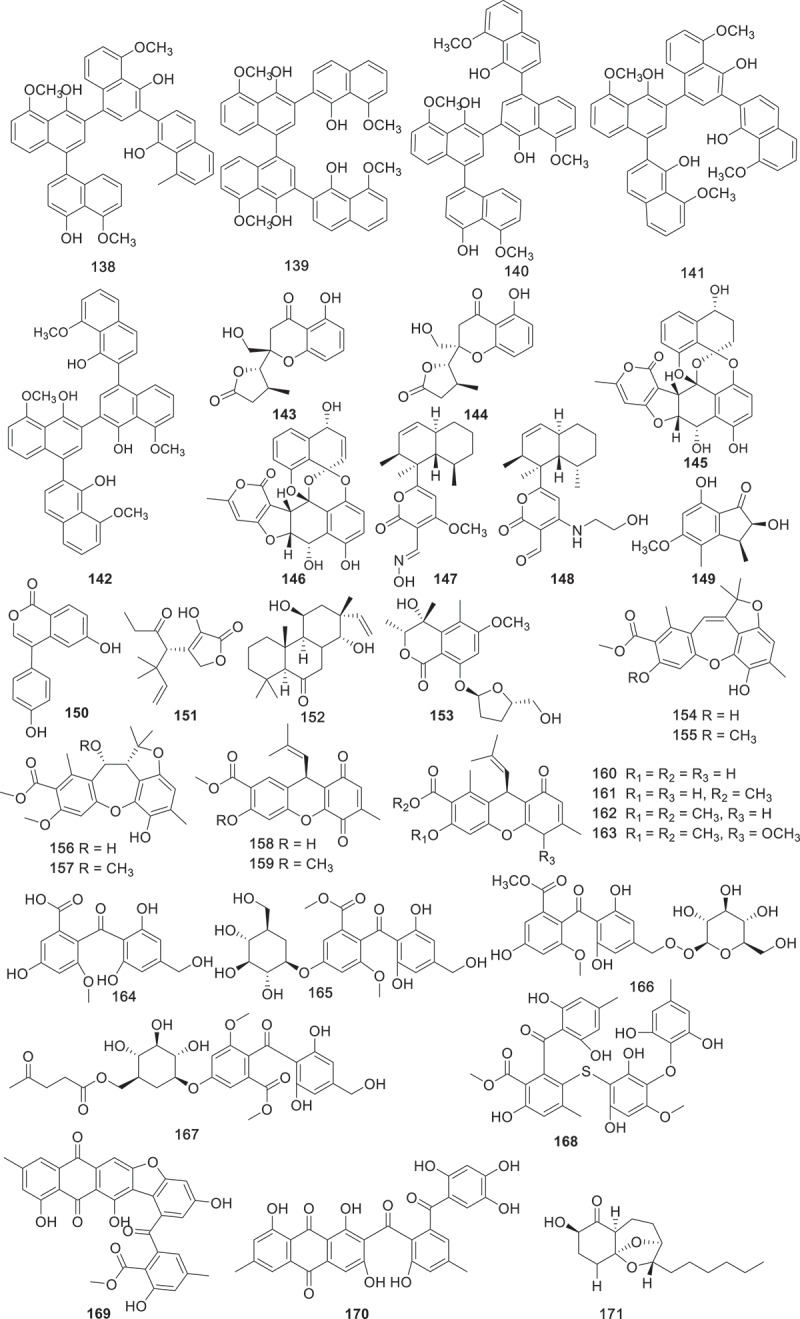
Structures of compounds **138**−**171**.

**Figure 8.**
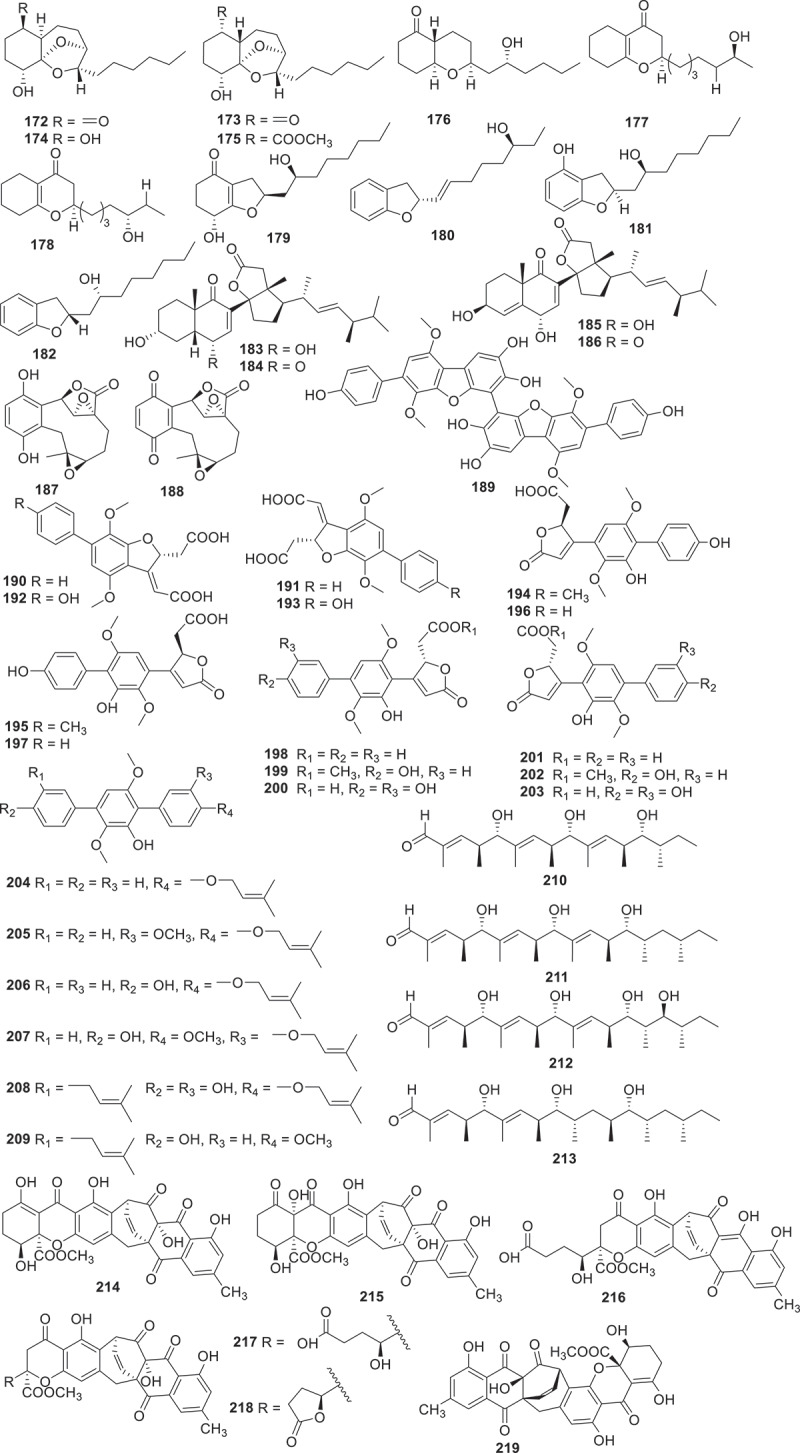
Structures of compounds **172**−**219**.

**Figure 9.**
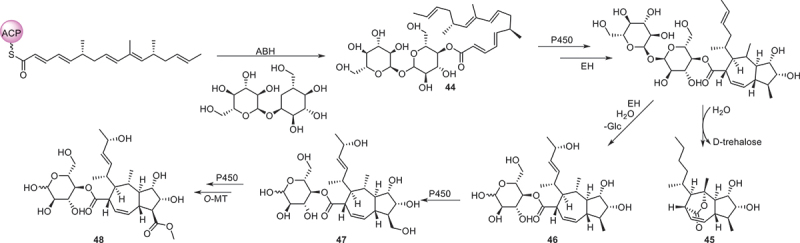
Biosynthetic pathway of compounds **44**−**48**.

**Figure 10.**
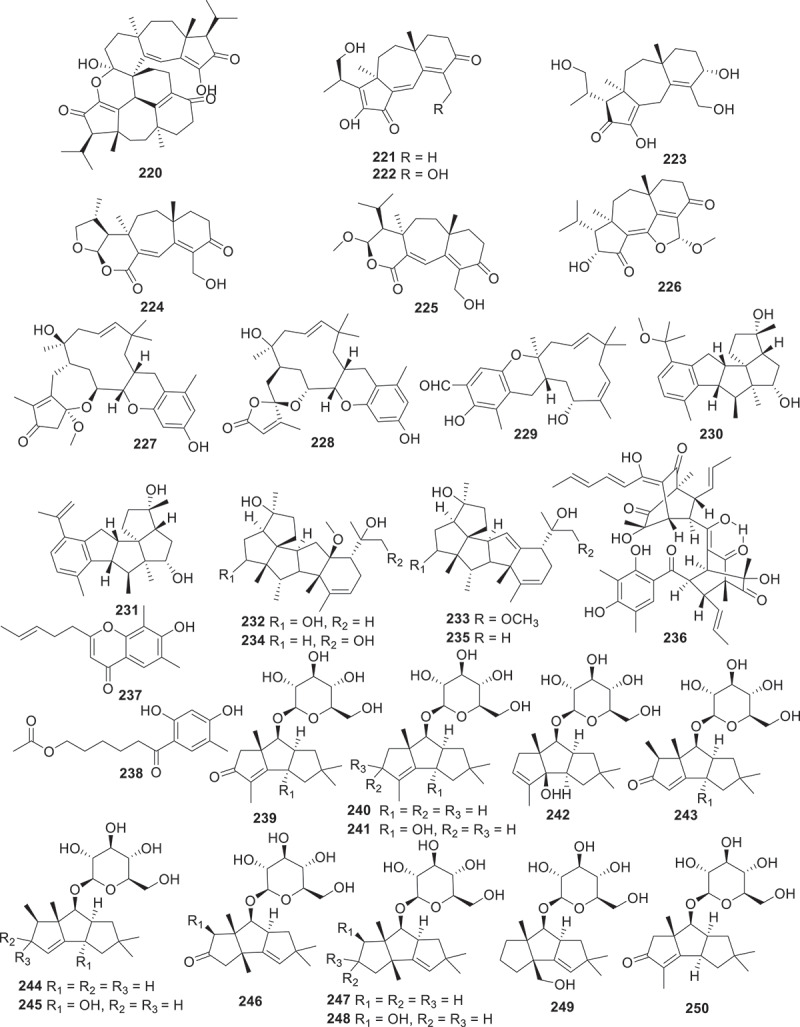
Structures of compounds **220**−**250**.

**Figure 11.**
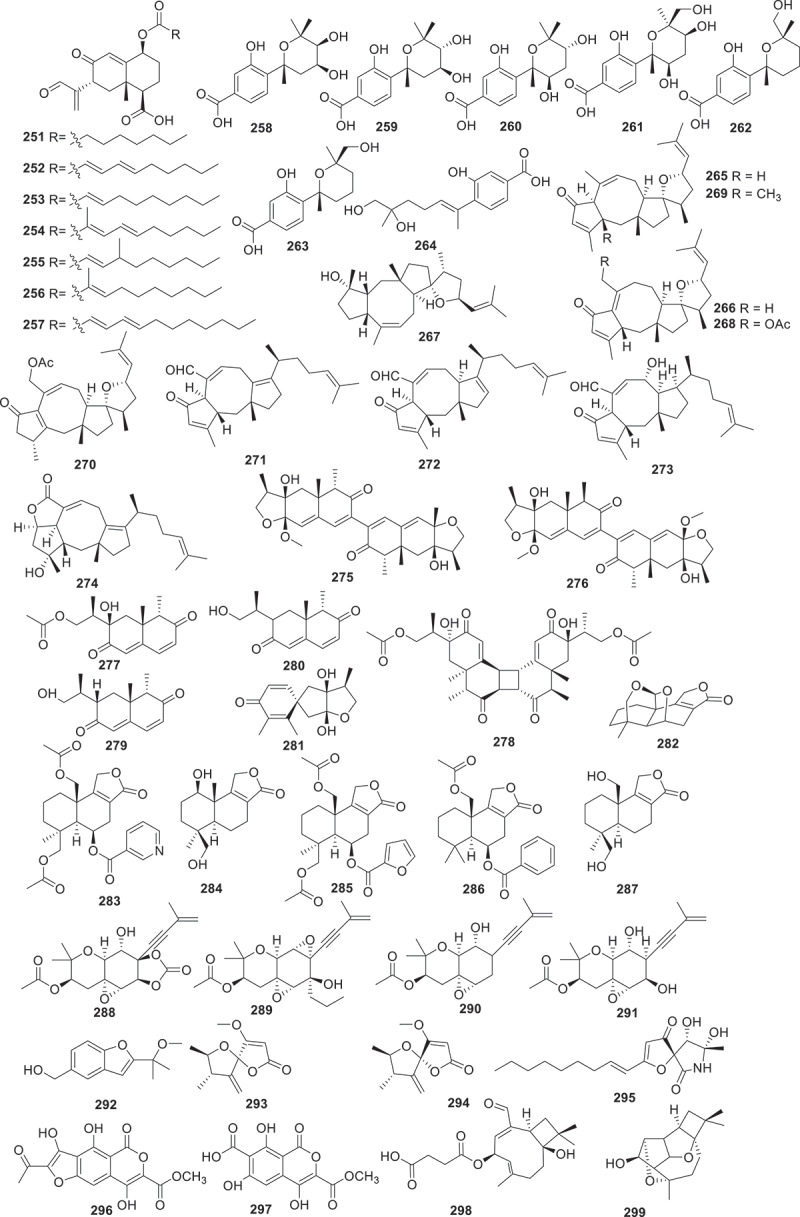
Structures of compounds **251**−**299**.

**Figure 12.**
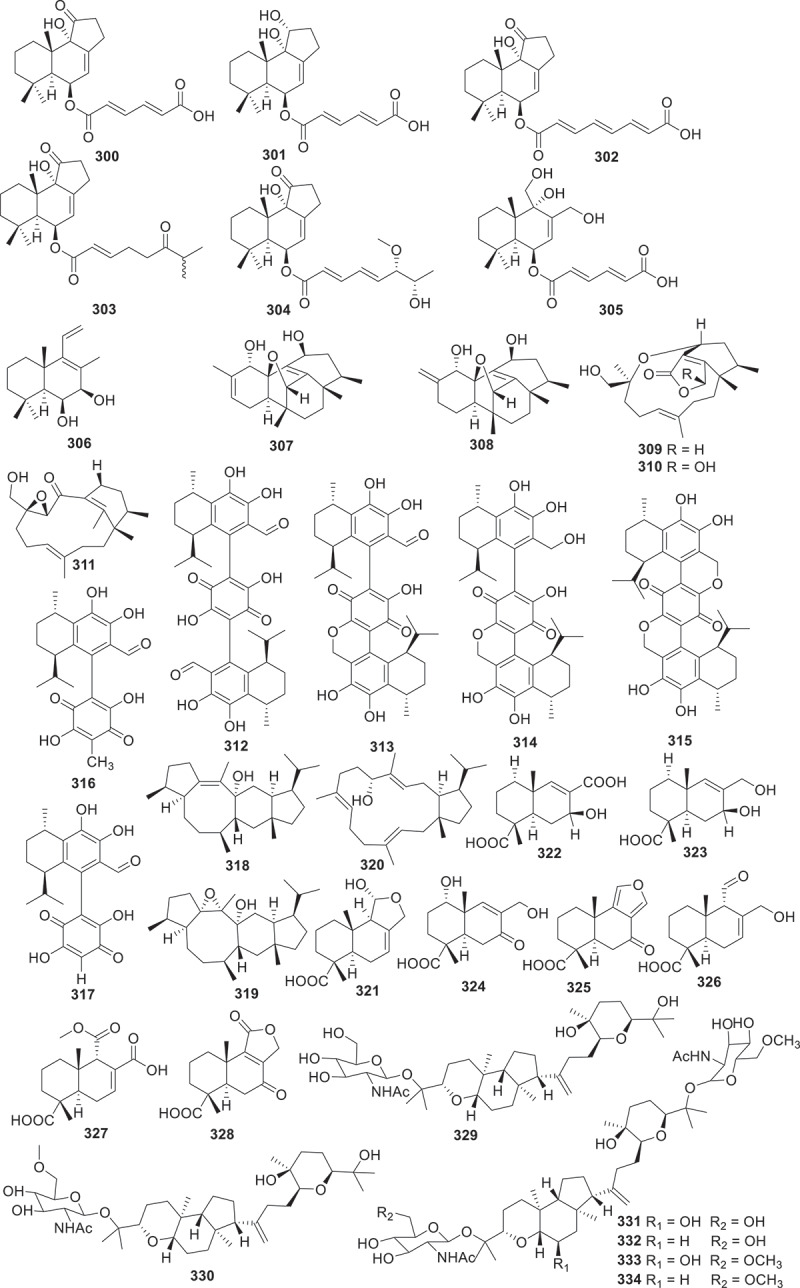
Structures of compounds **300**−**334**.

**Figure 13.**
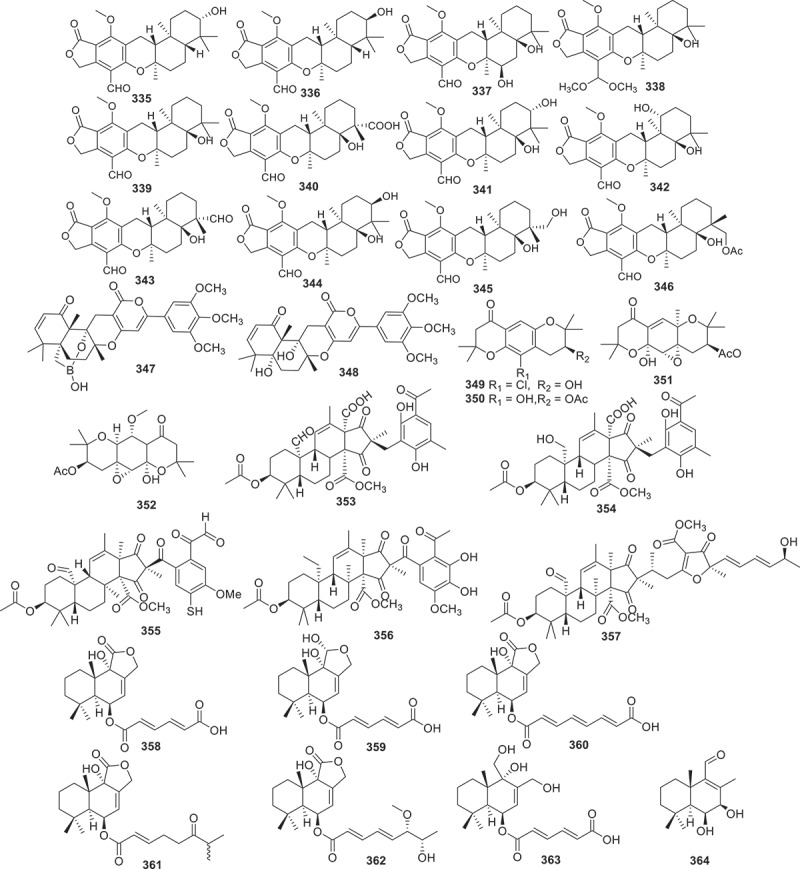
Structures of compounds **335**−**364**.

**Figure 14.**
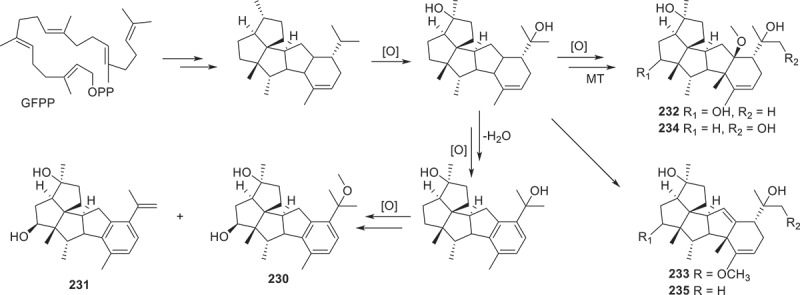
Biosynthetic pathway of compounds **230**−**235**.

**Figure 15.**
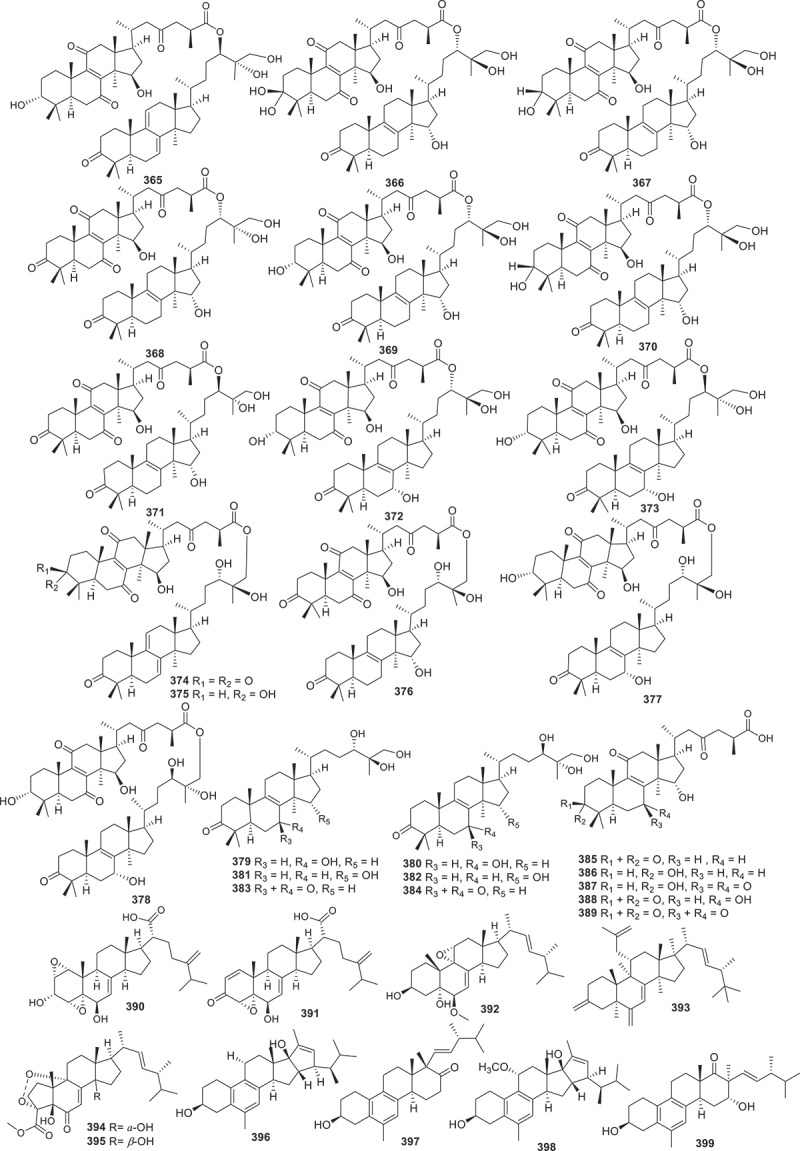
Structures of compounds **365**−**399**.

**Figure 16.**
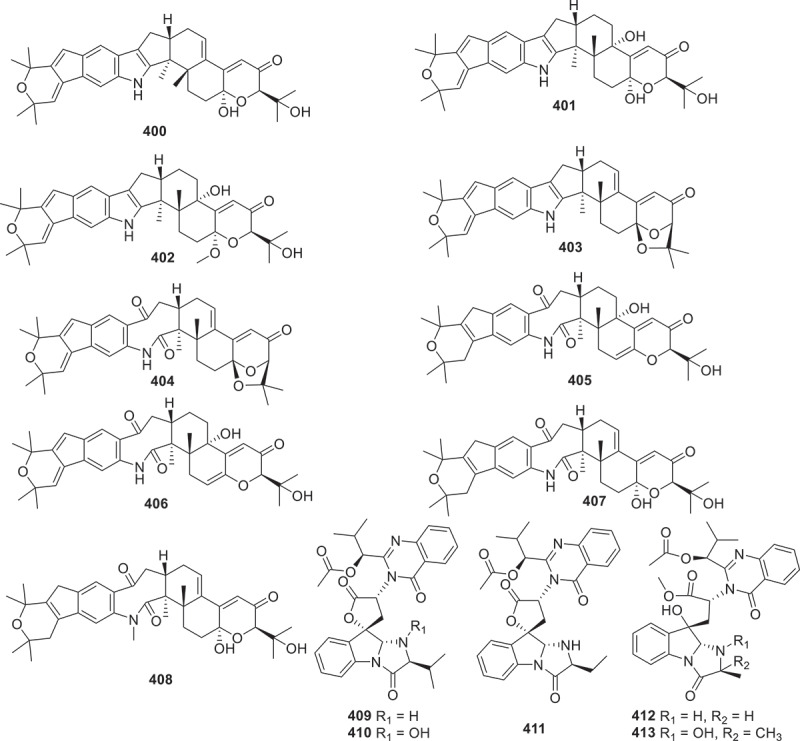
Structures of compounds **400**−**413**.

**Figure 17.**
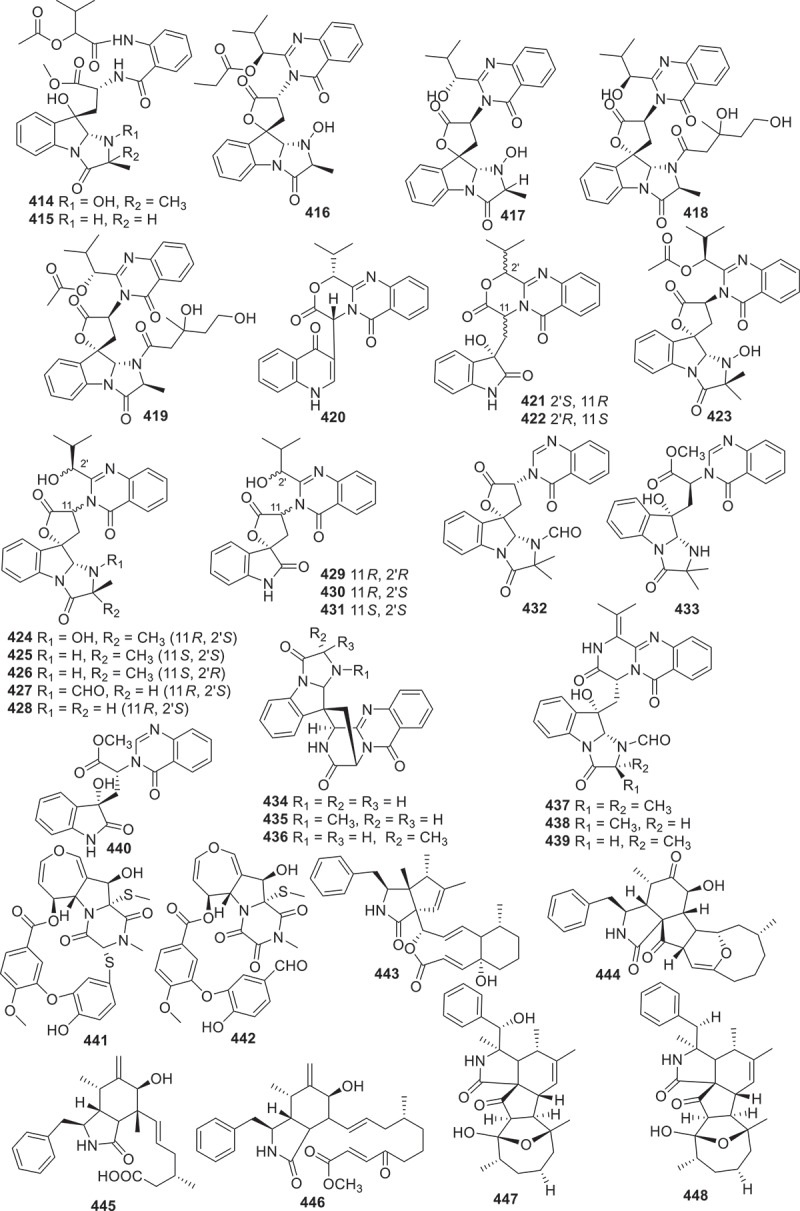
Structures of compounds **414**−**448**.

**Figure 18.**
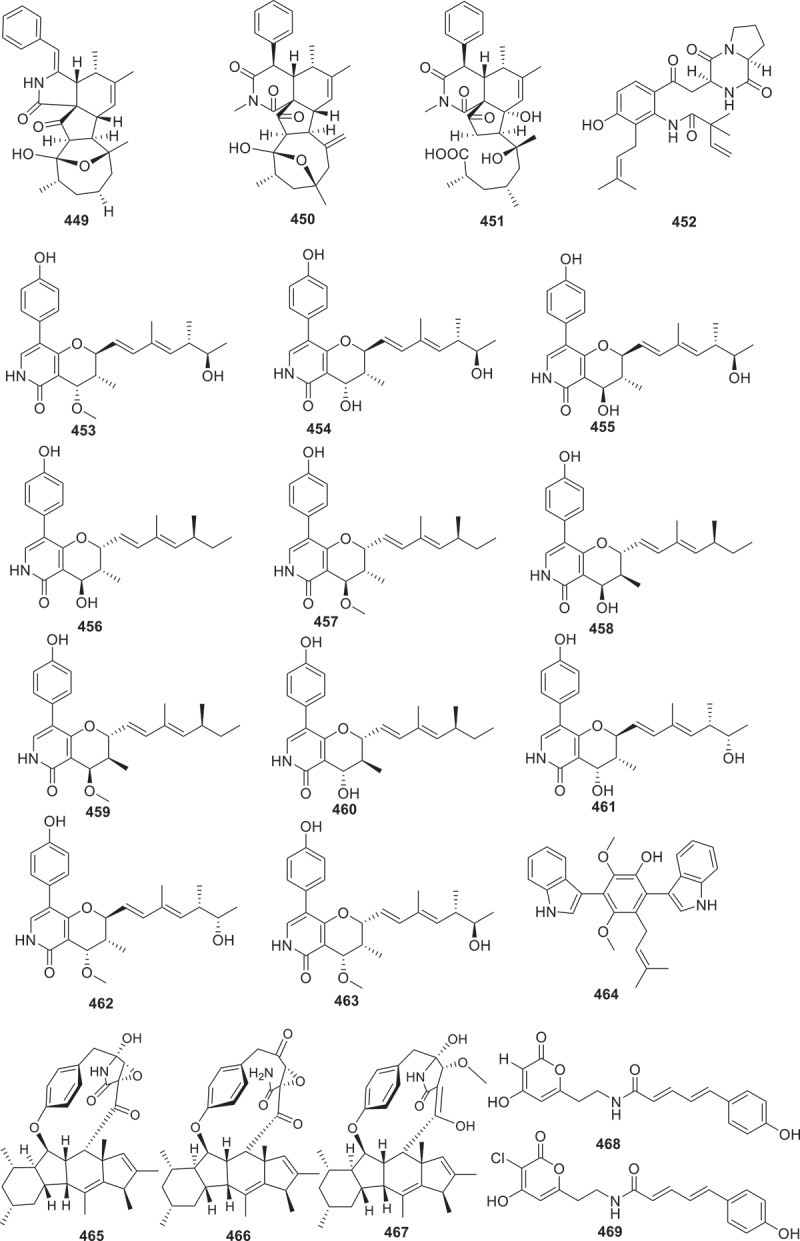
Structures of compounds **449**−**469**.

**Figure 19.**
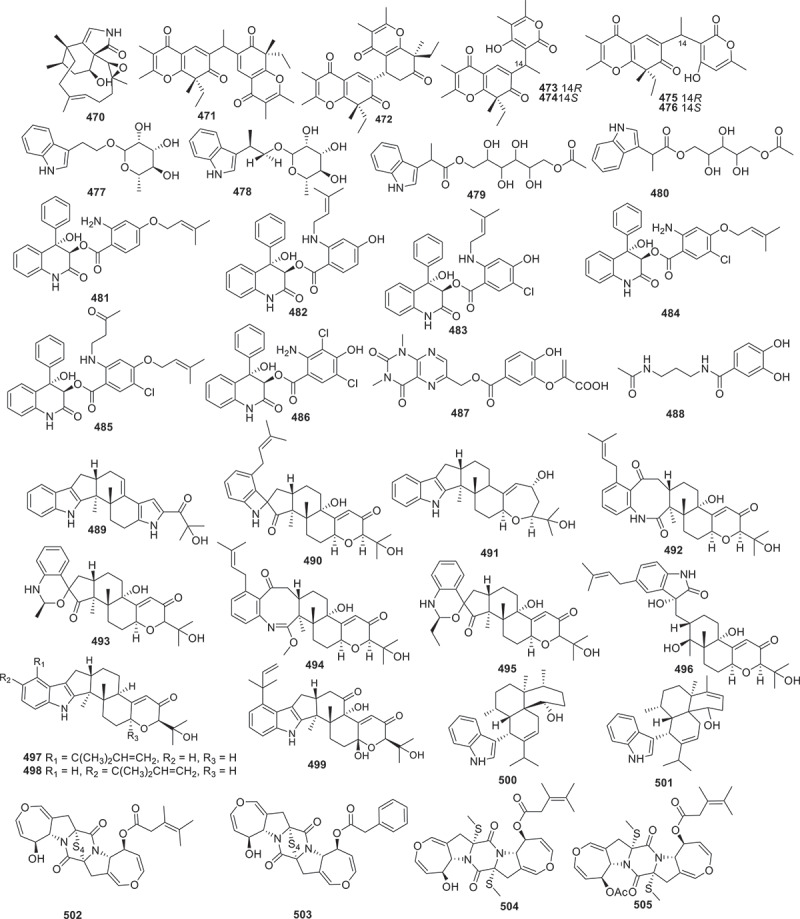
Structures of compounds **470**−**505**.

**Figure 20.**
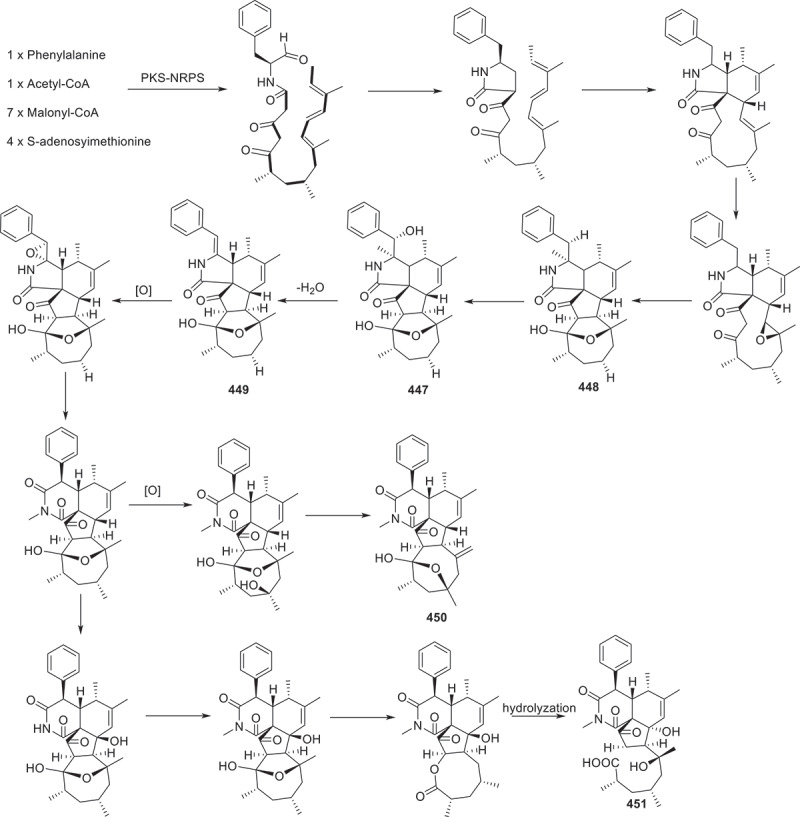
Biosynthetic pathway of compounds **447**−**451**.

Figure 2a gives an overview of new chemical structures from fungi reported in the literature published in 2023, categorised on the basis of their structural characteristics and their putative biogenetic origins. According to the related literature published in 2023, there are 553 new compounds derived from fungal secondary metabolites (structures are shown in Figures 3−20, including 219 polyketides, 145 terpenoids, 35 steroids, 106 alkaloids, and 48 peptides. Combined with the evidence from Figure 2a, polyketides play a dominant role, comprising 41% of all new natural products from fungi, followed by terpenoids at 26% (Figure 2a). Alkaloids accounted for the largest proportion of the remaining three structural types, reaching 20%, while steroids and peptides accounted for 7% and 6%, respectively.

Among the 553 new compounds isolated in 2023, less than half of them (212, approximately 40%) exhibited biological activities, which exposed another difficulty through natural products research as drug candidates due to the low amounts of biologically active natural products synthesised in very complex matrices. In the past year, researches have mainly focused on bioactivities including cytotoxic, anti-inflammatory, anti-bacterial, anti-fungal, anti-viral, anti-parasitic, and antioxidant as well as other effects. Among them, compounds with antibacterial activities occupy an advantage in number, followed by those with cytotoxic activities, including each structural type mentioned in the review. Furthermore, the estimation of listed bioactivities (Figure 2b) revealed that the majority of terpenoids exhibit cytotoxic activities, while the number of polyketides with antibacterial activities is the highest. Overall, polyketides are rich and diverse in structure and extensive in biological activity, establishing their priority in this review, followed by terpenoids, steroids, alkaloids, and peptides. Their structural characteristics, biological activities, and strain sources are systematically reviewed herein.

### 3.2. Polyketides

Fungal-derived polyketides and their hybrids play a significant role in expanding the chemical space of total natural products. Totally 219 polyketides (structures are shown in Figures 3–8) were summarised in this section. For instance, trilactones A−H (**1**−**8**) were isolated from previously unexplored strains of the fungus *Trichocladium crispatum* guided by molecular networking separation. Compounds **1** and **7** feature two unconventional bridged tricyclic core skeletons, while **2**, **3**, **5**, and **6** share a unique tetracyclic 9/5/6/6 ring system. Compound **4** exhibits an unusual 9/5/6/10/3-fused pentacyclic architecture, and **8** is a dimer connected by an unexpected C-C linkage. Additionally, the antiosteoporosis effects of compounds **1**−**8** were evaluated *in vivo* through a zebrafish model. The result suggested that trilactone G (**7**) significantly maintains bone formation-resorption homoeostasis with a comparable moderating effect to that of the positive control (alendronate) (Han et al. 2023).

Another series of unprecedented scaffolds, talarolactones A−G (**9**−**15**) were isolated from a soil-derived fungus *Talaromyces adpressus*. Compounds **9**−**15** are highly modified *α*-pyrone dimers containing a 4,7,7,8-tetrasubstituted 5,6,7,8-tetrahydro-2*H*-chromen-2-one. Furthermore, compounds **9**−**12** possess a novel spiro ring system. Compounds **13** and **14** exhibited potential inhibitory effects on the nitric oxide (NO) production, with IC_50_ values of 2.3 ± 0.1 and 3.7 ± 0.3 μmol/L, respectively, which were better than that of the positive control (dexamethasone, IC_50_ = 8.8 ± 0.7 μmol/L). Further mechanistic studies revealed that compounds **13** and **14** could reduce the inflammatory response in lipopolysaccharide (LPS)-induced RAW264.7 cells by blocking the nuclear translocation of nuclear factor *κ*B (NF-*κ*B) signal (Zheng et al. 2023). Two novel cyclopropane derivatives **16** and **17** along with seven previously unreported *α*-pyranone derivatives (**18**−**24**) were obtained from the marine-derived fungus *Stagonospora* sp. SYSU-MS7888. Compounds **16**, **17**, **23**, and **24** exhibited notable anti-inflammatory activities with IC_50_ values ranging from 3.6 to 22.8 μmol/L, which was better than that of the positive control indomethacin (IC_50_ = 26.5 ± 1.13 μmol/L) (Wu et al. 2023).

Three diphenyl ethers (**25**−**27**) and a cyclopentenone (**28**) were isolated from the fermentation broth of a marine derived fungus *Spiromastix* sp. SCSIO F190. Notably, compounds **25**−**28** exhibited strong activities against Gram-positive bacteria, including methicillin-resistant *Staphylococcus aureus* (MRSA), *Enterococcus faecalis* ATCC 29212 and *Bacillus subtilis* BS01, with minimum inhibitory concentration (MIC) values of 0.5 − 4 μg/mL (Cai et al. 2023). A study group from Japan had isolated five previously unreported integrasone derivatives (**29**−**33**) from *Lepteutypa* sp. KT4162, which was collected from a beech tree in Kochi prefecture, Japan. Biological assays revealed that **30** (IC_50_ = 2.5 ± 0.3 μmol/L) exhibited potential inhibition of HIV-1 integrase without cytotoxicity (Miura et al. 2023). Similarly, 10 novel anti-bacterial agents subplenones A−J (**34**−**43**) were isolated from the endophytic fungus *Subplenodomus* sp. CPCC 401465 guided by antimicrobial assays and molecular network-based analysis. All of the isolated compounds exhibited obvious inhibitory activities against Gram-positive bacteria. Particularly, compounds **34**, **38**, and **40** exhibited remarkable antibacterial effects against MRSA ATCC 700698 with a MIC value of 0.25 μg/mL. Moreover, these three compounds also showed potent anti-bacterial activities against vancomycin-resistant *Enterococcus faecium* (VRE) ATCC 700221 with the MIC values ranging from 0.5 to 1.0 μg/mL (Cai et al. 2023).

Using the genome mining strategy, researches focused on the BGCs of the fungus *Trichoderma afroharzianum* T-22, a novel highly reducing polyketide synthase (HRPKS) BGC was reported, while five new compounds **44**−**48** with trans-fused 5,7-bicyclic skeletons were isolated and identified. Further bioactive assay indicated that no antimicrobial or herbicidal activities were detected (Yan et al. 2023). Eight new phenalenones asperphenalenones F−M (**49**−**56**) were obtained from the fermentation extract of the endophytic fungus *Clonostachys rosea*. The structural analysis involved GC-MS analysis of hydrolysis products and determination of optical rotation identified the sugar constituent of glycosylated compounds **53**−**56** as *α*-D-mannose. This marks the first reported isolation of glycosides from the rice culture of the endophytic fungus *C. rosea*. Regarding the antibacterial efficacy, compounds **49**, **50**, and **52** exhibited significant activities against MRSA and *E. faecium*, and compound **49** demonstrated the highest potency against both bacterial strains with MIC values of 12.5 and 25 μmol/L, respectively (Yang et al. 2023). Three uncommon caged xanthone [6,6,6,6,6] polyketides daldipyrenones A−C (**57**−**60**) are discovered from an endolichenic fungus *Daldinia pyrenaica* 047188. Among them, daldipyrenone A (**57**) significantly enhanced adiponectin biosynthesis by two-fold compared to the positive control. These compounds were also evaluated for the antimelanogenic activities and the results showed that **57** displayed a strong antimelanogenic effect in the human melanoma MNT-1 cell lines (EC_50_ = 3.36 μg/mL), which was more potent than those of the positive controls arbutin (EC_50_ = 54.49 μg/mL) and kojic acid (EC_50_ = 66.65 μg/mL) (Lee et al. 2023).

Three new isocoumarins (**61**−**63**) together with one new pyrone derivative (**64**) were isolated from the fermentation broth of the mangrove endophytic fungus *Phomopsis* sp. DHS-11. Compounds **61** and **63** exhibited cytotoxic activities against HeLa cells with IC_50_ values of 11.49 ± 1.64 μmol/L and 8.70 ± 0.94 μmol/L, respectively. And **64** exhibited cytotoxic activity against human hepatoma cells HepG2 with an IC_50_ value of 34.10 ± 2.92 μmol/L (Guo et al. 2023). Moreover, inspired by OSMAC strategy, two novel dihydroisocoumarin glycosides xylarglycosides A (**65**) and B (**66**) were obtained from the fermentation of the fungus *Xylaria* sp. KYJ-15 on potato and rice solid media. Both compounds exhibited antibacterial activities against *Staphylococcus aureus* with MICs of 4 and 2 μg/mL, respectively, and showed 2,2-diphenyl-1-picrylhydrazyl (DPPH) radical scavenging activities comparable to the positive control with IC_50_ values of 9.2 ± 0.03 and 13.3 ± 0.01 μmol/L, respectively (Gan et al. 2023). Additionally, combined the OSMAC approach with molecular network-based untargeted metabolomics, two new PKS-NRPS hybrid macrolactone versicolide A (**67**) and quinazoline (−)-isoversicomide A (**68**) were isolated from the deep-sea fungus *Aspergillus versicolor* PS108-62 (Magot et al. 2023). Fruthermore, heterologous expression strategy was used in the investigation of the natural products derived from fungi. The xenoacremones BGC (PKS-NRPS) from the fungus *Xenoacremonium sinensis* ML-31 was successfully expressed in the *Aspergillus nidulans* host, leading to the identification of four novel tyrosine-decahydrofluorene analogs, named xenoacremones I−L (**69**−**72**) (Liu et al. 2023).

Seven new isocoumarins, named prunolactones A−G (**73**−**79**), featuring a unique 6/6/6/6/6/6 spiropentacyclic skeleton, were discovered from the endophytic fungus *Phomopsis prunorum*, guided by UPLC-QTOF-MS and^1^H NMR spectroscopic analytical techniques. Compounds **75**, **76**, and **79** exhibited significant pro-angiogenic activities in zebrafish at a concentration of 80 μmol/L (Dai et al. 2023). The fungus *Griseofulvania griseomyces* yielded six griseofulvin analogues leukomycins A−F (**80**−**85**), among which compounds **80** and **83** demonstrated promising anti-inflammatory properties in RAW264.7 macrophages and mice with ulcerative colitis (Liang et al. 2023). Chemical investigation of a lichen-associated fungus named *Phaeosphaeria* sp. SQ-510 had yielded six skeletally new dimeric spiciferones, phaeosphaerones A−F (**86**−**91**). Compounds **86** and **88**−**91** represented a novel class of chromene-pyrone hybrids featuring a unique ethylidene bridge. The plant-growth regulatory activity of these compounds was evaluated, and the results demonstrated that compounds **86**, **87**, **90**, and **91** inhibited the growth of the weed-like dicot on *Arabidopsis thaliana* at concentrations of 100 μmol/L. Further studies showed that low concentrations of compound **86** promoted the growth of *A. thaliana* by increasing fresh weight and/or root elongation, while significant inhibition was observed at high concentrations (Zhai et al. 2023).

Sixteen novel polyketides, ophicirsins A−P (**92**−**107**) were discovered from the extract of the endophytic fungus *Ophiobolus cirsii* LZU-1509. Compounds **96**−**100** and **105**−**107** encompass novel carbon frameworks, while **105** and **106** feature different cyclic ether connected with an aromatic ring system. The antioxidant effects of the isolated compounds were further evaluated using the model of oxidative damaged neuron-like PC12 cells, and compound **106** exhibited excellent protective capacity (nearly rescuing the cell viability completely) for hydrogen peroxide insult. Furthermore, compound **106** displayed a remarkable capacity to scavenge the free radicals of DPPH (Guo et al. 2023). Seven novel tanzawaic acid derivatives, steckwaic acids E−K (**108**−**115**), and a novel benzene derivative (**115**) were extracted from a marine-derived fungus *Penicillium steckii* SCSIO 41040. Compound **109** was found to suppress the nuclear effect induced by LPS and exhibited inhibitory activity against LPS-induced NF-*κ*B with IC_50_ value at 10.4 μmol/L (BAY11-7082 was used as a positive control, IC_50_ = 5.0 μmol/L). Furthermore, compound **109** could suppress the RANKL-induced osteoclast differentiation in bone marrow macrophage cells (BMMCs) triggered by NF-*κ*B ligand (RANKL) (Song et al. 2023).

Four structurally unique paraphaeolactones A_1_, A_2_, B_1_, and B_2_ (**116**−**119**) were isolated from the culture broth of *Paraphaeosphaeria* sp. KT4192. None of these compounds showed notable antifungal activity against *Bipolaris oryzae* (*Cochliobolus miyabeanus*). The WST-1 assay using COLO 201 human colon adenocarcinoma revealed weak cytotoxicities of **118** and **119**, with IC_50_ values of 260 and 200 μmol/L, respectively (Kanehara et al. 2023). Assisted by a molecular networking-based strategy for natural product dereplication and prioritisation, three unprecedented 1-deoxy-D-glucosamine adducts, glyclauxins A−E (**120**−**125**), were obtained from a nest-derived fungus *Talaromyces* sp. CMB-MW102. The author proposed a biogenetic relationship linking all members of the extended duclauxin structure class. However, none of their biological activities were evaluated (Samarasekera et al. 2023). Through genome mining and heteroexpression, a HRPKS gene cluster (*cpn*) from *Calcarisporium arbuscula* was successfully identified and activated. Heterologous expression of the *cpn* cluster in the engineered *Aspergillus nidulans* A1145 host led to the isolation of four new *α*-pyrone compounds calcapyrones A−D (**126**−**129**). The *cpn* cluster encodes a HRPKS (*cpn*A), an *O*-methyltransferase (*cpn*B), and a cytochrome P450 (*cpn*C). First, the HRPKS *Cpn*A synthesised a reduced polyketide chain from one molecule of acetyl-CoA and five molecules of malonyl-CoA to generate intermediate. Then this intermediate is converted to intermediates **128** and **129** catalysed by the *O*-methyltransferase *Cpn*B. Lastly, the cytochrome P450 *Cpn*C catalysed the regioselective dihydroxylation of intermediates **128** and **129** to form compounds **126** and **127**, respectively. It was found that compound **128** showed modest cytotoxic effects against HCT116, HepG2, and B16 cell lines, with IC_50_ values of 21.7 ± 0.7, 41.8 ± 0.7, and 38.5 ± 1.0 μmol/L, respectively, while the positive control (cisplatin) showed IC_50_ values of 20.8 ± 0.8, 19.3 ± 0.6, and 22.5 ± 1.5 μmol/L, respectively (Dong et al. 2023). A new terrein derivative aspergilethers A (**130**) was obtained from the endophyte *Aspergillus terreus* HT5. Notably, structural analysis revealed that **130** had a unique medium aliphatic side chain. However, no phytotoxic activity was detected on **130** (Wang et al. 2023).

Six unreported compounds ustilagols A−F (**131**−**136**) were discovered from the pathogenic fungus *Ustilago maydis* MZA96986, and compound **133** (IC_50_ = 6.7 ± 0.5 μmol/L) along with **135** (IC_50_ = 5.8 ± 0.9 μmol/L) showed effective inhibition of NO production in microglia BV-2 cells lines (Wu et al. 2023). Additionally, six previously unreported naphthol tetramers (**137**−**142**), including naphthol monomers, dimers and trimers, were isolated from *Daldinia childae* 047219 using feature-based molecular networks. However, none of these new compounds exhibited anti-inflammatory activity against NO production (Kim et al. 2023). Two new dihydrochromones, paecilins Q (**143**) and R (**144**), were isolated from the endophytic fungus *Pseudofusicoccum stromaticum* CMRP4328. **143** strongly inhibited the growth of mycelium of plant pathogen *Phyllosticta citricarpa* with low cytotoxic activity, which deserves further investigation for the control of citrus black spot disease (Iantas et al. 2023). Two previously unreported preussomerin derivatives, lasiodiplodiapyrones A and B (**145** and **146**), were isolated from the soil-derived fungus *Lasiodiplodia pseudotheobromae* 414-JZ-40. **145** and **146** are adducts of an *α*-pyrone and a polyketide. Further study on the anti-inflammatory activities of the isolated compounds indicated that both compounds could decrease LPS-induced NO production with IC_50_ values of 4.8 ± 0.3 and 8.5 ± 1.1 μmol/L, respectively, with MG132 (IC_50_ = 2.6 ± 0.3 μmol/L) as the positive control (Liang et al. 2023). Seven new polyketides (**147**−**153**) were isolated from the crude extract of the marine-derived fungus *Peroneutypa* sp. M16. The strain was revealed under the guidance of antiplasmodial screening and processing attractive antiplasmodial activity. Compound **151** had potent activity against chloroquine-sensitive and antiplasmodial strains (IC_50_ = 19 − 37 μmol/L) without cytotoxic effect at a concentration as high as 100 μmol/L (Oliveira et al. 2023).

Ten new diphenyl ether-polyketides rhoxocerins A−D (**154**−**157**) and rhoxocercosporins A−F (**158**−**163**) were isolated from endophytic fungus *Rhexocercosporidium* sp. Dzf14. Compounds **154**−**157** represent a new tetracyclic carbon skeleton (6/7/5/6). The results of the antibacterial assay showed that compounds **154**, **158**, and **160**−**162** displayed potent activities against MRSA (MIC = 16, 32, 16, 16, and 4 μg/mL, respectively), with vancomycin (MIC = 2 μg/mL) as the positive control. In addition, compounds **154**, and **158**−**162** exhibited remarkable activities against VRE (MIC = 16, 8, 8, 16, 8, and 4 μg/mL, respectively). Furthermore, these compounds were also tested for cytotoxic activities, but no activities were found (Gu et al. 2023). Seven new aromatic polyketides (**164**−**170**) were obtained from the fermentation extract of Antarctic moss-derived fungus *Pseudogymnoascus* sp. OUCMDZ-3578. The inhibitory activities of the isolated compounds against beta amyloid protein A (A*β*_42_) aggregation were investigated by thioflavin T assay. According to the results, compounds **169** and **170** (IC_50_ = 0.10 μmol/L, 0.18 μmol/L) showed the highest inhibitory activity of A*β*_42_ accumulation, equivalent to the positive drug epigallocatechin gallate (EGCG) (IC_50_ = 0.14 μmol/L). Intensive study found that compound **170** had a dominant disaggregation effect on Fe^3+^ induced A*β*_42_ aggregates (Yin et al. 2023). Twelve novel polyketides, koningiopisins I−P (**171**−**178**) and trichoketides C−F (**179**−**182**) were isolated from the fungus *Trichoderma koningiopsis*. Compounds **171**−**175** are tricyclic polyketides with an octahydrochromium skeleton with a 6,8-dioxadicyclic [3.2.1] octane core. Compounds **177** and **178** contain a unique ketocarbonyl group on C-7, unlike other compounds that contain ketocarbonyl groups on C-1. Compounds **171** (IC_50_ = 14 ± 1 μmol/L) and **172** (IC_50_ = 3.0 ± 0.5 μmol/L) inhibited LPS-induced NO production in BV-2 cells, respectively (Huang et al. 2023).

Four new 9,11-secosteroid-derived *γ*-lactones altersteroids A−D (**183**−**186**) were isolated from cultures of the ascomycete fungus *Aternaria* sp. All of the compounds were tested for cytotoxicities against four tumour cell lines. Compound **185** showed moderately cytotoxicity against four types of tumour cells (IC_50_ = 4.8−12.7 μmol/L) and induced apoptosis of A549 cells, with a more potent effect than the positive control cisplatin (1.6−25.9 μmol/L) (Fu et al. 2023a). Harms et al. discovered that the extracts of the basidiomycete *Resupinatus* sp. BCC84615 exhibited activity against *Bacillus subtilis*, which was screened for further chemical investigation. Two novel compounds **187** and **188** were obtained from the culture of this fungus. Both of them exhibited antibacterial activities on *B. subtilis* and *S. aureus* at the concentration of 17 μg/mL, and showed weak cytotoxicities against cancer cell lines (Harms et al. 2023). Under the guidance of molecular networking, 21 new *p*-terphenyl derivatives, asperterphenyls (**189**–**209**), were obtained from a sponge-derived fungus *Aspergillus* sp. SCSIO41315. These compounds were evaluated their neuraminidase inhibitory activities and antiviral effects. Compound **189** displayed neuraminidase inhibitory activity with an IC_50_ value of 1.77 ± 0.53 μmol/L (Wei et al. 2023).

Linear polyketides scytalpolyols A−D (**210**−**213**) were isolated from the *Scytalidium* sp. IQ-074. The inhibitory activities of compounds **210**−**213** against *h*PTP1B_1–400_ were evaluated. Scytalpolyol B (**211**) could significantly inhibit *h*PTP1B_1–400_ with an IC_50_ value of 27.0 ± 1.7 μmol/L, comparable to that of the positive control ursolic acid (IC_50_ = 26.6 μmol/L). This was the first report of a polyenol that acts as an inhibitor of *h*PTP1B_1–400_ (Martinez et al. 2023). A fungal extract from *Trichocladium* sp. TN09213 RBM-1 was found to inhibit four infectious disease-causing organisms *Mycoplasma genitalium*, *Plasmodium falciparum*, *Cryptosporidium parvum*, and *Trichomonas vaginalis* with low toxicity to human liver cells. The intensive chemical investigation led to the separation of six new compounds, named xanthoquinodins NPDG A_1_–A_5_ (**214**−**218**) and B_1_ (**219**). Unfortunately, none of these new compounds were identified as key factors contributing to the antimicrobial activity of crude extracts (Lee et al. 2023).

According to the above analysis, bioactive polyketides with anti-bacterial activity accounted for the largest proportion. For example, compounds **25**−**28**, **34**, **38**, **40**, **49**, **50**, **52**, **65**, **66**, **143**, **154**, **158**, and **160**−**163** possessed remarkable activities against both human and plant pathogen bacteria with a comparable effect to that of the positive control. Additionally, compounds **13**, **14**, **16**, **17**, **23**, and **24** exhibited outstanding anti-inflammatory activities, while compounds **61**, **63**, **64**, **118**, **119**, **128**, and **185** displayed significant cytotoxic activities. Furthermore, the biosynthetic pathway of compounds **44**−**48** (Figure 9) is selected as a case of biosynthesis of polyketides. The BGC producing compounds **44**−**48** encodes a set of homologous enzymes, including an HRPKS (TreA), an *α*/*β* hydrolase (ABH), a P450 monooxygenase (P450), an *α*-glucosidase, a protein predicted to be a terpene cyclase (TC) but having sequence homology to epoxide hydrolases (EHs), and two additional conserved genes encode a P450 and an *O*-methyltransferase (*O*-MeT). Coexpression of TreA with the ABH led to the accumulation of compound **44**, and subsequent biosynthesis of compounds **45**−**48** is further catalysed by P450, *O*-MeT, EHs, etc (Yan et al. 2023).

### 3.3. Terpenoids

Terpenoids, characterised by the isoprene units in their structure, include various subclasses such as sesquiterpenes, diterpenes, and triterpenes. Within this classification, a total of 145 terpenoids are described in detail in this section (Figures 10−13).

Seven new guanacastane diterpenoids, named psayamin (**220**), psathins A−F (**221**−**226**) were produced by the culture of *Psathyrella candolleana* in host *Dioscorea opposite* medium. Among these new structures, compound **220** featured a novel 5/7/6/6/6/6/7/5-fused octacyclic spiro scaffold and exhibited cytotoxic activity against five tumour cells HL-60, A549, SMMC-7721, MCF-7, and SW480 with the IC_50_ values ranging from 10.87 ± 0.24 to 15.96 ± 0.30 μmol/L (Zhao et al. 2023). Two tropolonic meroterpenoids with unprecedented pentacyclic skeletons phomaketals A (**227**) and B (**228**), together with pughiinin B (**229**) were obtained from the culture of the fungus *Phoma* sp. CGMCC 10481. Biogenetically, compounds **227** and **228** are assumed to originate from different reaction cascades of the hypothetical tropolonic sesquiterpene intermediates neosetophomone B and 9-*R*-neosetophomone B. Compound **227** displayed antiproliferative effect specifically against the SUPB15 cells, with an IC_50_ value of 4.85 μmol/L (Li et al. 2023). Niduenes A–F (**230**−**235**) are six functionalised sesterterpenoids isolated from the endophytic fungus *Aspergillus nidulans*. These compounds feature a unique 5/5/5/5/6 pentacyclic ring skeleton, with **230** and **231** representing the first examples of aromatic pentacyclic sesterterpenoids. Compound **233** showed potent resensitisation of SW620/AD300 cells to paclitaxel (PTX). Further mechanistic studies revealed that compound **234** inhibitory to the efflux function of P-glycoprotein (P-gp) (Fu et al. 2023b).

Using the method of liquid chromatograph-mass spectrometer (LC-MS) analysis to screen endophytes from the traditional Chinese medicinal herb *Coptis chinensis* Franch., the fungus *Trichoderma citrinoviride* HT-9 has been identified. Further chemical investigation led to the isolation of a novel homotrimeric sorbicillinoid, citrinsorbicillin A (236), together with two new monomers, citrinsorbicillins B (**237**), and C (**238**). Compound **236** represented a unique carbon skeleton and exhibited moderate inhibitory activity against human colon cancer H-29 cells (Yin et al. 2023). Antrodizonatins A−L (**239**−**250**) are previously undescribed triquinane sesquiterpene glycosides isolated from the fruiting bodies of *Antrodiella zonata*. All the compounds were evaluated for their antibacterial activities against *S. aureus*, *E. coli*, *Pseudomonas aeruginosa*, and *Salmonella enterica*. Among them, compounds **239**, **243**, and **250** exhibited weak inhibitories against the growth of *S. aureus* with IC_50_ values of 35, 34, and 69 μmol/L, respectively (Gao et al. 2023). Based on *in vitro* antibacterial activity assays against human pathogenic bacteria, a bioactivity-guided fractionation workflow led to the purification of seven novel bioactive eremophilane sesquiterpenes, eremoxylarins D−J (**251**−**257**), from an endolichenic fungus *Xylaria hypoxylon* cultivated in coculture with another endolichenic fungus *Dendrothyrium variisporum*. Compounds **251**, **253**, **254**, and **256** showed selective activities against MRSA, with MIC values ranging from 0.39 to 12.5 μg/mL. Particularly, eremoxylarin I (**256**) exhibited the strongest and broadest antibacterial activity among the isolated compounds, and it also exhibited activity against HCoV-229E (IC_50_ = 18.1 μmol/L) (Miral et al. 2023).

During the investigation of the marine-derived fungus *Aspergillus versicolor* YPH93, seven new phenolic bisabolane sesquiterpenoids (**258**−**264**) were discovered. Compound **264** selectively inhibited ferroptosis with EC_50_ values ranging from 2 to 4 μmol/L. However, **264** did not show any effect on TNF*α*-induced necroptosis or H_2_O_2_-induced cell necrosis. Additionally, compound **264** exhibited negligible free radical scavenging activity in an antioxidant-DPPH assay (Zheng et al. 2023). Using LC-MS/MS-based molecular networking strategy combined with bioactive evaluation, 10 undescribed analogues bipolaricins J−S (**265**−**274**) were isolated from the phytopathogenic fungus *Bipolaris eleusines* ACCC30957. Compounds **271**−**273** displayed strong inhibitory activities against B16 cells, with IC_50_ values of 8.05 ± 2.46, 4.51 ± 0.84, and 3.53 ± 0.62 μmol/L, respectively (*cis*-platinum was used as a positive drug, IC_50_ = 10.86 ± 0.94 μmol/L). Additionally, compounds **265**, **268**−**273** also exhibited inhibitory effects against HepG2, with IC_50_ values ranging from 1.15 ± 0.33 to 74.64 ± 12.00 μmol/L (*cis*-platinum was used as the positive drug, IC_50_ = 9.15 ± 1.27 μmol/L). Furthermore, compounds **266**−**273** showed significant effects against MCF-7 cell lines with IC_50_ values ranging from 9.27 ± 1.03 to 35.04 ± 2.84 μmol/L (*cis*-platinum was used as a positive drug, IC_50_ = 15.58 ± 0.88 μmol/L) (Wei et al. 2023). Seven new compounds named paraconulones A−G (**275**−**281**) were isolated from the marine-derived fungus *Paraconiothyrium sporulosum* DL-16. Except for compound **275**, all of the other compounds exhibited a strong inhibitory effects on LPS-induced NO production in BV2 cells, with IC_50_ values ranging from 2.8 ± 0.5 to 98 ± 17 μmol/L, comparable to the positive control curcumin (IC_50_ = 8.6 ± 1.6 μmol/L) (Sun et al. 2023). By applying the strategy of OSMAC throughout the chemical investigation of an isopod-derived fungus *Aspergillus parasiticus* SDU001, six compounds including astellolide R (**282**), featuring an unusual cage-like 6/6/5/6/5 pentacyclic ring system, astellolide S (**283**) and astellolides T−W (**284**−**287**) were isolated. Remarkably, astellolide S (**283**) is the first fungal drimane-type sesquiterpenoid that integrates a nicotinic acid moiety at C-6. Furthermore, compounds **284** and **287** showed moderate anti-inflammatory activity by inhibiting the LPS-induced NO production in RAW264.7 macrophages with IC_50_ values of 6.1 ± 0.8 and 6.8 ± 0.8 μmol/L, respectively (dexamethasone was used as the positive control, IC_50_ = 2.6 ± 0.3 μmol/L) (Dai et al. 2023). By utilising a combination of ^13^C NMR and biosynthetic information strategies to investigate the secondary metabolites from the fungus *Amphichorda felina* SYSU-MS7908, five new compounds acetylenic meroterpenoids felinoids A−E (**288**−**292**) were obtained. Compound **288** is a rare cyclic carbonate in a noteworthy natural acetyl terpenoid compound. Compounds **288**−**292** showed anti-inflammatory activities by inhibiting the production of NO in LPS-induced RAW264.7 cells (IC_50_ = 11.6−19.5 μmol/L), with the indomethacin as the positive control (IC_50_ = 35.8 ± 2.5 μmol/L) (Jiang et al. 2023b). Additionally, 7-deoxypapyracillic acids A and B (**293** and **294**) were isolated from the *Scytalidium* sp. IQ-074 (Martinez et al. 2023). Three new compounds, miktospiromide A (**295**), kitrinomycins A (**296**), and B (**297**) were obtained through the application of cocultivation of the fungi *Penicillium brasilianum* MST-FP1927 and *Aspergillus nomius* MST-FP2004. During subsequent bioactivity evaluation, **296** demonstrated a significant inhibitory effect on mouse melanoma NS-1 cells, with a median lethal concentration (LD_99_) of 7.8 μmol/L. Furthermore, **296** displayed inhibitory activity against bovine parasite *Tritrichomonas foetus*, with LD_99_ value of 4.8 μmol/L (Cowled et al. 2023).

Two new caryophyllene sesquiterpenes punctaporonins T (**298**) and U (**299**) were yielded from the fungus *Chaetomium globosum*. Compound **298** exhibited selective activity against *Mycobacterium tuberculosis* H37Ra and *S. aureus*, with IC_50_ values of 105 μmol/L and 237 μmol/L, respectively (Morehouse et al. 2023). Seven new terpestacin-type sesterterpenoids maydistacins A−G (**300**−**306**) were isolated from the phytopathogenic fungus *Bipolaris maydis*. Compound **300**, a terpestacin derivative with a unique bicyclic fused ring system, exhibited inhibition of NO production in LPS-induced RAW264.7 macrophages with an IC_50_ value of 19 ± 2 μmol/L. It is the first terpestacin-type sesterterpenoid reported to display anti-inflammatory effects (Shi et al. 2023).

The deep sea-derived fungus *Paraconiothyrium hawaiiense* FS482 was shown to produce five novel diterpenoids hawanoids A–E (**307**–**311**). The structures of these compounds were elucidated through X-ray crystallographic analysis and NMR spectroscopy. These compounds were tested for inhibitory effects on platelet activating factor (PAF)-induced platelet aggregation. Compounds **309** and **310** showed significant activities with IC_50_ values of 7.1 and 8.9 μmol/L, respectively, while compounds **307**, **308**, and **310** exhibited moderate activities with IC_50_ values ranging from 15−67 μmol/L (Chen et al. 2023).

New meroterpenoids bis-heimiomycins A−D (**312**−**315**) and hemimycins D and E (**316** and **317**), were isolated from the *Heimiomyces* sp. MUCL 56078. These compounds were all tested for cytotoxicities against the human cervical cancer cell lines KB3.1 and the murine fibroblast cell lines L929. Only compound **316** exhibited a cytotoxic effect against KB3.1 cells with an IC_50_ of 6.3 μmol/L. In addition, **316** displayed cytotoxic effect against other cell lines including breast cancer cell lines MCF-7 (IC_50_ = 2.5 μmol/L), ovarian cancer cell lines SKOV-3 (IC_50_ = 3 μmol/L), and skin cancer cell line A431 (IC_50_ = 4.25 μmol/L) (Pfutze et al. 2023). Three new compounds were isolated from a fungal *Alternaria alternata* MB-30 symbiont coevolved with sesterterpenoid-producing plants. Their structures were identified as a 5/8/6/5 tetracyclic sesterterpenoid, sesteraltererol (**318**), together with its absolute stereochemistry 10,11-epoxysesteraltererol (**319**) and a 5/15 bicyclic sesterterpenoid preterpestacin I (**320**), respectively. These compounds showed inhibitory effects on lipid accumulation during adipocyte differentiation (Li et al. 2023). Abundisporin A (**321**), together with seven monoterpenoids, named abundisporins B−H (**322**−**328**), were obtained from *Abundisporus violaceus* MUCL 56355. Although the isolated compounds exhibited neither significant antimicrobial nor cytotoxic activities, they demonstrated noteworthy neurotrophic effects in promoting nerve growth. Particularly striking was the finding that when subjected to 5 ng/mL of nerve growth factor (NGF), **321** and **326** were observed to significantly enhance neurite growth (Sum et al. 2023). From the culture of *Onygenales* sp. YX1425, researchers identified six novel squalene-derived polyether glycosides, named onygenaleosides A−F (**329**−**334**). These compounds exhibit a unique chemical structure characterised by a 6/5 bicyclic fused ring skeleton. Subsequent bioactivity assessments revealed that compound **332** demonstrated weak efficacy against the *Spodoptera frugiperda*, displaying a median lethal concentration (LC_50_) of 193.4 ± 1.1 µg/mL (Chen et al. 2023).

Twelve new austalide meroterpenoids, diaporaustalides A−L (**335**−**346**), were isolated from the endophytic fungus *Diaporthe* sp. XC1211. **336** and **340** showed potent proliferation inhibitory effects against LPS-induced B cells, with IC_50_ values of 6.7 and 3.8 μmol/L, respectively. Additionally, both compounds decreased the secretion of IL-6 in LPS-induced B cells in a dose-dependent manner (Chang et al. 2023). A drimane meroterpenoid borate, named territrem F (**347**), along with its diol precursor territrem B (**348**), were isolated from the fungus *Alternaria* sp. ZH-15 associated with the soft coral. These compounds featuring a unique borate ring system showed potential as synchronous Ca^2+^ oscillation inhibitors. Both compounds showed significant inhibitory activities on spontaneous synchronous Ca^2+^ oscillations (SCOs) and epileptic discharges induced by 4-amino-pyridine (Wang et al. 2023). Jiang et al. (2023c) discovered that the culture extract of the fungus *Amphichorda felina* SYSU-MS7908 displayed moderated cytotoxicity against U87-MG human glioma cells. Then the isolation of four new meroterpenoids amphicordins A−D (**349**−**352**) had been reported. Compound **351** possessed a rare benzo[*g*]chromene (6/6/6) skeleton. However, none of these compounds showed cytotoxic effects. Five new meroterpenoids peniandranoids A−E (**353**−**357**) were yielded from the culture extract of the fungus *Penicillium* sp. sb62. Compound **353** displayed remarkable inhibitory activities towards influenza virus A (H1N1) with an EC_50_ value of 19 μg/mL, while **355**−**357** showed immunosuppressive activities against concanavalin A-induced T cell proliferation with EC_50_ values ranged from 4.3 to 27 μmol/L and LPS-induced B cell proliferation with EC_50_ values ranging from 7.5 to 23 μmol/L (Chang et al. 2023).

Seven new drimane-type sesquiterpenoids, named drimanenoids A−G (**358**−**364**) were isolated from the ethyl acetate extract of the earwig-derived *Aspergillus* sp. NF2396. Structurally, drimanenoids A−G (**358**−**364**) are new members of drimane-type sesquiterpenoid esterified with unsaturated fatty acid side chain at C-6. Compounds **360**, **361**, and **362** showed antibacterial activities against five types of bacteria (*Xanthomonas oryzae* pv. *oryzae*, *Xanthomonas campestris* pv. *Mangiferaeindicae*, *Escherichia coli*, *Micrococcus luteus*, and MRSA) with different inhibition diameters. **362** exhibited moderate cytotoxicity against human myelogenous leukaemia cell line K562 with an IC_50_ value of 12.88 ± 0.11 μmol/L (Salman et al. 2023).

To sum up, terpenoids possessed both various chemical diversity and significant biological activities. As mentioned in this section above, compounds **220**, **227**, **233**, **236**, **265**−**273**, **316**, and **362** exhibited outstanding anti-tumour activities against several cell lines, while compounds **360**, **361**, and **362** possessed excellent antibacterial activity. Additionally, the biosynthetic pathway of compounds **230**−**235** (Figure 14) is reviewed to represent the biosynthesis of terpenoids. The initial head-to-tail cyclisation of geranylfranesyl pyrophosphate (GFPP) and Wagner-Meerwein alkyl with hydride shift produce 5/5/5/5/6 pentacyclic ring intermediates, subsequent oxidation and methylation reactions afford compounds **230**−**235** (Fu et al. 2023b).

### 3.4. Steroids

Steroids are a class of natural products characterised by a distinctive cyclopentano-perhydrophenanthrene carbon skeleton. This section reviews 35 new steroids derived from fungi in 2023 (Figure 15).

Investigation of the fungus *Ganoderma weberianum* yielded 11 unreported lanostane dimers including ganoweberianones C−H (**365**−**370**) and isoganoweberianones A (**371**), B (**372**), D (**373**), G (**374**), and H (**375**), together with six new ganodermanontriol derivatives as three pairs of diastereomers (**376**/**377**, **378**/**379**, and **380**/**381**). Additional five new ganoweberianic acids, ganoweberianic acids H–L (**382**−**386**), were also isolated. **371** and **373** showed significant antimalarial activities against *Plasmodium falciparum* K1 (multidrug-resistant strain) with IC_50_ values of 0.057 and 0.035 μmol/L, respectively, while their cytotoxicities against Vero cells were weak (IC_50_ = 8.1 and 19 μmol/L, respectively) (Chinthanom et al. 2023).

Nine new ergosteroids (**387**−**395**) were isolated from the fungal strain *Talaromyces adpressus*. The cytotoxic activities of compounds **387**−**395** were evaluated against five human cancer cell lines (HL-60, SD-DHL-2, PKO, HepG2, and A549). **388**−**392** and **395** exhibited cytotoxic activities with IC_50_ values ranging from 0.4 to 32 μmol/L. Compound **393** showed an immunosuppressive effect against LPS-induced B lymphocyte proliferation with an IC_50_ value of 8.6 μmol/L (Zheng et al. 2023). Five new aromatic ergosterols with unique ring systems spectasterols A−E (**396**−**399**) were obtained from the culture extract of the fungus *Aspergillus spectabilis*. Compounds **396** and **398** possess a 6/6/6/5/5 ring system with an additional cyclopentene, while **397** and **399** contain an uncommon 6/6/6/6 ring system. According to the results of their bioactivities, **397** possessed cytotoxic activity against SU-DHL-2 cells and HL60 cells with IC_50_ values of 6.9 μmol/L and 8.7 μmol/L, respectively (Wei et al. 2023).

Overall, steroids derived from fungi exhibited obvious bioactivities. For instance, compounds **371** and **373** not only displayed significant antimalarial activities against *Plasmodium falciparum* K1 (multidrug-resistant strain) but also showed cytotoxicities against Vero cells. Compounds **388**−**392** and **395** exhibited cytotoxic against HL-60, SD-DHL-2, PKO, HepG2, and A549 cell lines, while compound **397** possessed cytotoxic activity against SU-DHL-2 cells and HL60 cells. Unfortunately, none of the literature mentioned above reported the biosynthetic pathways of the isolated compounds.

### 3.5. Alkaloids

Alkaloids are a class of natural organic compounds that contain nitrogen atoms. This review summarises totally 106 new alkaloids (Figures 16–19) from fungi associated secondary metabolites.

Co-culturing *Penicillium janthinellium* with *Paecilomyces formosus* resulted in the discovery of nine new indole-diterpenes, named janthinellumines A–I (**400**–**408**). These compounds exhibited a wide range of biological activities, including anti-influenza A virus, protein tyrosine phosphatase (PTP) inhibitory effects, and anti-Vibrio activities. Specifically, compound **403** displayed significant activity against the strains A/WSN/33 (H1N1) and A/Hong Kong/1/68 (H3N2), with IC_50_ values of 3.8 μmol/L and 13.3 μmol/L, respectively. Compounds **400**, **403**, **404**, and **406**–**408** also showed activities against both strains, with IC_50_ values ranged from 7.3 μmol/L to 20.6 μmol/L (Cao et al. 2023). Eleven new indole quinazoline alkaloids aspergillus A–K (**409**–**419**) were discovered from the culture extract of the fungus *Aspergillus clavatonanicus*, which was collected from the gut of centipedes. The myocardial cell protective activities of these compounds were determined, and the results revealed that compounds **409**, **410**, and **413** could improve the damage caused by cold ischaemia (CI) within 48 hours after CI, and compounds **410** and **413** could also prevent GSK3*β* induced by cold ischaemia at 12 hours after CI dephosphorylation of Ser9 (Jin et al. 2023). A group of indoloquinazoline alkaloids named clavutoines A–U (**420**–**440**) were isolated from the marine-derived fungus *Aspergillus clavutus* LZD32-24. The inhibitory effects against anti-angiogenesis and cytotoxic activities towards human umbilical vein endothelial cells (HUVECs) were evaluated. However, none of the compounds displayed any effects (Guo et al. 2023).

Two novel alkaloids noremestrin A (**441**) and secoemestrin E (**442**) were isolated and characterised from the extract of the fungus *Emericella* sp. 1454. Preliminary biological activity tests were conducted on these two compounds. Compounds **441** and **442** exhibited weak cytotoxicities against human chronic myeloid leukaemia cell lines K562, with IC_50_ values of 63.6 and 15.3 μmol/L, respectively. Both compounds also showed weak cytotoxicities against MEG-01 cell lines, with IC_50_ values of 71.1 and 23.0 μmol/L, respectively (Chen et al. 2023). Four unprecedented cytochalasins boerelasins A–D (**443**–**446**) were isolated from the endophytic fungus *Boeremia exigua*. These compounds exhibited cytotoxic activities against five human cancer cell lines, including HL-60, A549, SMMC-7721, MCF-7, and SW480. Compound **445** showed potent cytotoxicity against HL-60, SMMC-7721, and MCF-7 cells, with IC_50_ values of 2.89 ± 0.13, 4.33 ± 0.16, and 5.79 ± 0.07 μmol/L, respectively. Compounds **443**, **444**, and **446** showed moderate cytotoxicities against some or all of the cancer cell lines, with IC_50_ values ranging from 9.73 to 30.56 μmol/L (Shi et al. 2023).

At the same time, metabolic analysis of the endophytic fungus *Chaetomium nigricolor* F5 was conducted and five new cytochalasins featuring a novel 5/6/5/5/7-fused pentacyclic skeleton, chamisides B–F (**447**–**451**) were discovered. Furthermore, these compounds were tested on *Arabidopsis thaliana* root elongation models. Compound **447** showed moderate inhibition on root elongation (Gu et al. 2023). A bioactive indole alkaloid amoenamide D (**452**) was isolated from *Aspergillus amoenus* TJ507 collected from the leaves of *Hypericum wilsonii*. Compound **452** was found to improve liver ischaemia/reperfusion injury. The experimental results indicated that this compound could reduce hepatocyte apoptosis and liver damage, as well as lower the levels of alanine aminotransferase (ALT), aspartate aminotransferase (AST), and lactate dehydrogenase (LDH) in serum. In addition, this compound could reduce the expression of myeloperoxidase (MPO) in liver tissue and alter the localisation of high mobility group protein B1 (HMGB1) (Zhang et al. 2023). The marine-derived fungus *Penicillium oxalicum* QDU1 was investigated for the production of interconvertible pyridone alkaloids. The researchers isolated and identified 11 new compounds penicipyridones A−K (**453**−**463**). The biological activities of these compounds were evaluated. None of them exhibited anti-bacterial or anti-fungal activity. However, compounds **453**, **456**, **457**, **460**, **461**, and **463** showed moderate inhibition of NO production by RAW264.7 macrophages, with IC_50_ values ranging from 9.2 to 19 μmol/L (Wu et al. 2023).

A new anthraquinone compound ochrindole F (**464**) was extracted from an endophytic fungus *Aspergillus* sp. GZWMJZ-258, isolated from the fruit body of *Garcinia multiflora*. Observation on the growth of human acute myeloid leukaemia (AML) cell lines MV 411 and human normal liver cell lines L-02, compound **464** showed cytotoxic effect against MV 411 cells (IC_50_ = 1.8 μmol/L) and L-02 cells (IC_50_ = 17 μmol/L) (Wang et al. 2023). Three new enbesin analogues (**465**−**467**) were obtained from the culture extract of the fungus *Sarocladium* sp. MSX6737 by bioactivity-directed isolation. **465**−**467** belong to macrocyclic alkaloids with a cyclopentadiene[*b*]fluorene ring system, and showed cytotoxicities against human breast cancer cell lines (MDA-MB-231), with IC_50_ values ranging from 0.4 to 4.8 μmol/L. Compound **467** also showed cytotoxic activity against human ovarian cancer cells (OVCAR3) and MDA-MB-435 cells, with IC_50_ values of 1.0 and 1.8 μmol/L, respectively (Al Subeh et al. 2023).

Two avenualamide pyranone compounds fuligopyrones A and B (**468** and **469**) were extracted from the fungus *Fuligo septica*. Although these two compounds have no biological activity, they have unique mechanisms and effects against UV radiation, providing short-term protection and reducing abiotic stress caused by UV radiation (Minns et al. 2023). Moreover, the first nitrogen-containing phytoactin phytoactinine (**470**) was isolated from marine fungus *Biatriospora* sp. CBMAI 1333. At concentrations of 10 and 25 μmol/L, **470** could inhibit cPAF induced IL-8 generation (Oliveira et al. 2023). A novel class of chromene-pyrone hybrids named phaeosphaerones A–F (**471**–**476**), were isolated from the fungus *Phaeosphaeria* sp. Compounds **471** and **473**–**476** possess a unique ethylidene bridge. The plant-growth regulatory activities of compounds **471**–**476** were evaluated using two herbaceous plants *Arabidopsis thaliana* and *Oryza sativa*. The results demonstrated that these compounds exhibited effects in promoting plant growth, with glyphosate and glufosinate used as positive controls in the study (Zhai et al. 2023). Furthermore, four new plant growth inhibitory compounds, colletotriauxins A–D (**477**–**480**) were discovered from the phytopathogenic fungus *Colletotrichum gloeosporioides* NRRL 45420. These compounds inhibited the growth of *Lepidium sativum* seedlings, with the inhibition of stem growth being stronger than IAA, particularly with compounds **479** and **480** being the most effective. This indicated that **477**–**480** may be promising herbicide candidates (Zhou et al. 2023).

Eight novel alkaloids containing benzoic acid named asperalins A–F (**481**–**486**), asperalumazine A (**487**), and *N*-(3-acetamidopropyl)-3,4-dihydroxyben-zamide (**488**), were discovered from a seagrass derived fungus *Aspergillus alabamensis*. **481**–**488** showed inhibitory activities against fish pathogenic bacteria, including *Edwardsiella ictaluri*, *S. iniae*, and *S. parauberis*. **483** and **484** were particularly effective against *S. aureus*, *S. inia*e, and *S. parauberis*. Compound **485**, a derivative of **484**, had the strongest inhibitory effects on *S. iniae*. Compound **486** exhibited inhibitory effects against all tested strains. Compound **487** is the first lumazine derivative directly linked to a benzoic acid moiety (Hu et al. 2023). The strain *Penicillium* sp. DG23, isolated from Chinese traditional medicine *Schisandra macrocarpa*, was characterised by producing eight novel indole diterpenoids schipenindolenes A−H (**489**−**496**). Compound **489** showed potent activity as an HMG-CoA reductase (HMGCR) degrader (Su et al. 2023). Five new indole diterpenoids asppaxillines A–E (**497**−**501**) were reported from the fungus *Nigrospora* sp. under the guidance of molecular networking. **497**−**501** displayed significant eliminating activities against chloroquine-sensitive strains (*P.f*.3D7) with IC_50_ values ranging from 0.84 to 2.9 μmol/L (Yang et al. 2023). From the culture extract of the fungus *Exophiala mesophila* MCCC 3A00939, four new compounds named graphamines K−N (**402**−**505**) were discovered. Among these compounds, graphamines K (**502**) and L (**503**) contain unusual bridging tetrathionyl groups. Additionally, compounds **502**, **504**, and **505** represented the first examples to contain the 3,4-dimethylpenton-3-enoate group at C-9. Investigating the bioactivities of these compounds revealed that **502** and **503** exhibit cytotoxic effects against various cancer cell lines (K562, H69AR, and MDA-MB-231), with IC_50_ values ranging from 2.3 to 5.9 μmol/L (Cheng et al. 2023).

In summary, the number of alkaloids with cytotoxic activity accounted for the largest proportion in this section. For instance, compounds **445**, **465**−**467**, **502**, and **503** displayed potent cytotoxicity against HL-60, SMMC-7721, MCF-7, MDA-MB-231, OVCAR3 K562, and H69AR cell lines. Additionally, potential antiplasmodial agents **497**−**501** displayed significant eliminating activities against chloroquine-sensitive strains (*P.f*.3D7). Apart from the bioactivies, the biosynthetic pathway of compounds **447**−**451** (Figure 20) is taken as an example of the biosynthetic process of alkaloids. Compounds **447**−**449** were derived from a widely accepted PKS-NRPS biosynthetic precursor composed of an octaketide and a phenylalanine. Furthermore, **447**−**449** might be the key biosynthetic precursors of co-isolated compounds **450** and **451**, enabling researches to revisit and update the previously proposed biosynthesis of cytochalasans with a piperidine-2,6-dione ring, such as **450** and **451** (Gu et al. 2023).

### 3.6. Peptides

**Figure 21.**
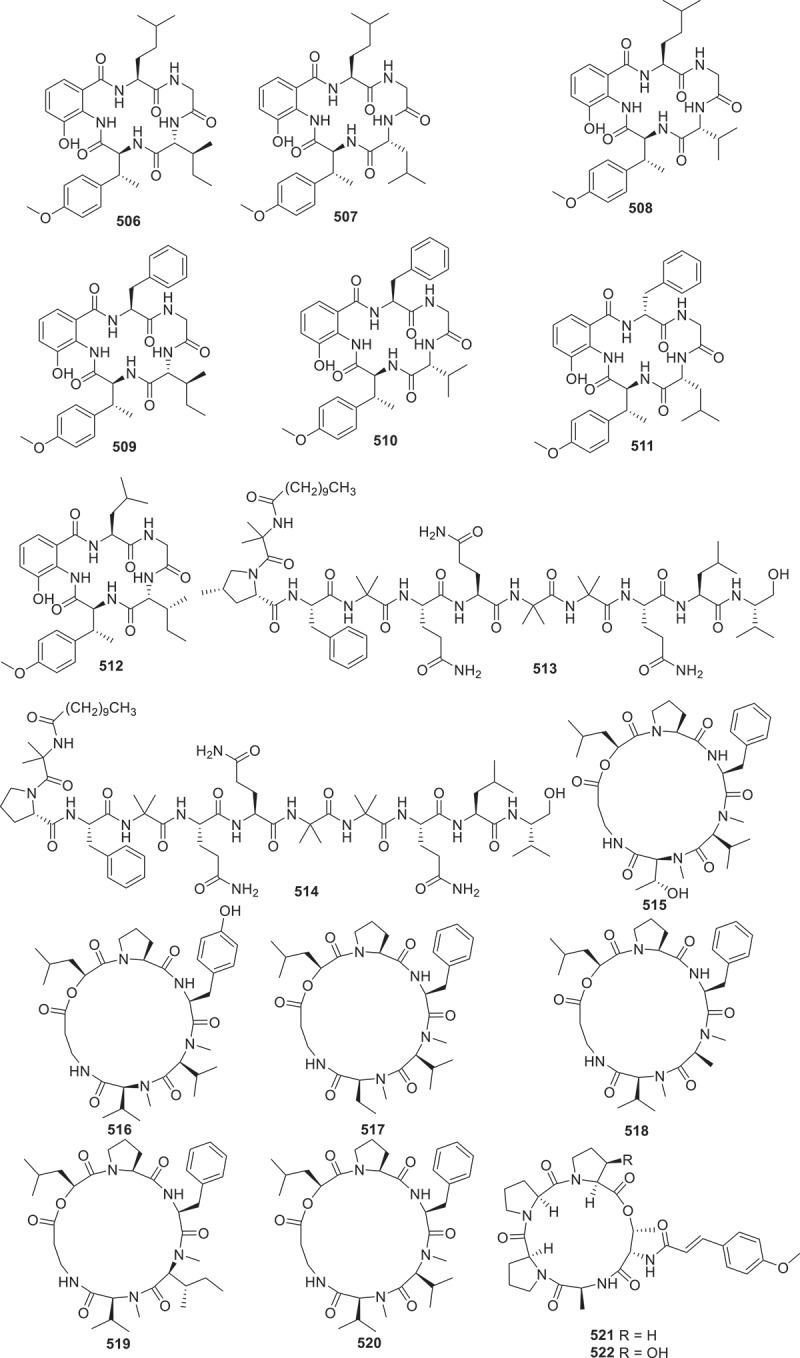
Structures of compounds **506**−**522**.

**Figure 22.**
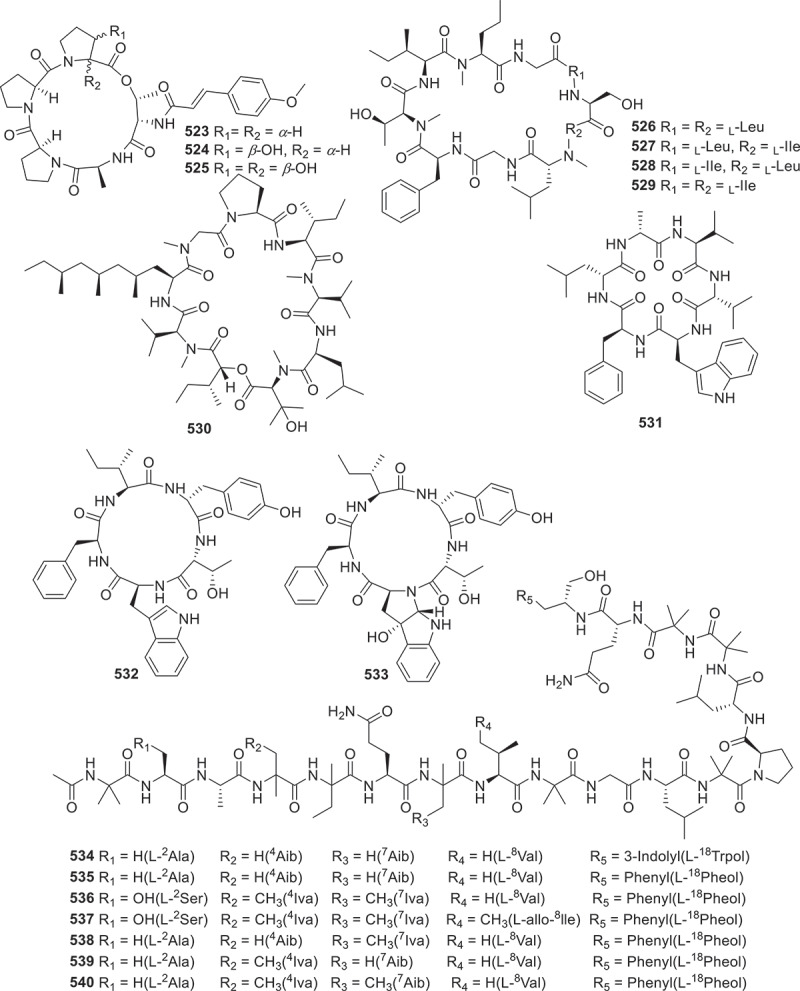
Structures of compounds **523**−**540**.

**Figure 23.**
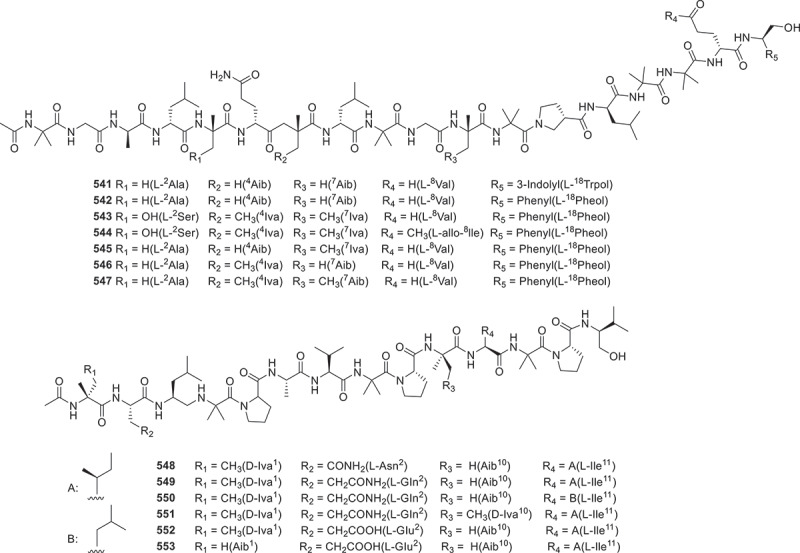
Structures of compounds **541**−**553**.

This section contains 48 new peptides derived from fungi metabolites (Figures 21−23). For instance, molecular networking-guided isolation of the fungus *Aspergillus pseudoviridinutans* TW585 resulted in the separation of seven novel cyclic pentapeptides pseudoviridinutans A−F (**506**−**512**). These compounds contain the rare amino acid fragment, which has been discovered for the first time in marine-derived fungi. Among them, compound **513** exhibited significant anti-inflammatory effects by inhibiting LPS-induced production of NO (Ding et al. 2023). Tolypocaibolas A (**513**) and B (**514**) are two newly discovered compounds isolated from marine-derived fungus *Tolypocladium* sp. Both compounds displayed moderate selective inhibitions against Gram-positive and acid-fast strains, while maximomycin [(P/M)-3)] exhibited moderate broad-spectrum antibacterial activity (Morehouse et al. 2023).

From the antagonistic metabolites produced by the fungus *Beauveria felina* isolated from marine ascidias, six novel cyclic peptides isaridins I−N (**515**−**520**), were identified. Furthermore, activity tests exhibited that **516** and **517** had significant inhibitory effects on the growth of *Geotrichum citri-aurantii* mycelia with the EC_50_ value of 56.8 ± 3.5 μg/mL, with triadimeon (146.4 ± 13.4 μg/mL) as the positive control (Jiang et al. 2023a). Moreover, five rare pentaphosphopeptide compounds aspertides A−E (**521**−**525**), were discovered to share a distinctive *p*-methoxycinnamamide group. These compounds were initially isolated from deep sea derived fungus *Aspergillus insuetus* SD-512. Compounds **524** and **525** exhibited notable antibacterial properties against a spectrum of bacteria including *Edwardsiella tarda*, *Vibrio alginolyticus*, *V. angularis*, *V. vulnificus*, and *S. aureus*, with MIC values ranging from 8 to 32 μg/mL (Chi et al. 2023). Four poly-methylated cyclodecapeptides (**526**−**529**) were identified from a culture of the fungus *Sesquicillium* sp. QL0466, which exhibited *in vitro* growth inhibitory activities against vancomycin-resistant *Enterococcus faecalis*, with MIC values of 8 μg/mL (Xiao et al. 2023).

Persephacin (**530**) was derived from the endophytic fungus *Elsinoe* sp. based on the conducting bioactivity tests. It was found that compound **530** demonstrated significant antifungal properties against *Aspergillus fumigatus* (Du et al. 2023). Three novel cyclic peptides meristosporins A−C (**531**−**533**) were successfully isolated and identified from *Basidiobolus meristosporus* Drechsler, a facultative parasitic fungus found in soil. These compounds were found to contain amino acid residues that are uncommon in this organism. Subsequent bioactivity experiments revealed that **531** and **532** exhibited significant cytotoxic effects on RAW264.7 macrophages and 293T renal epithelial cells (Zhao et al. 2023). The fungus *Trichoderma* sp. GXIMD 01001 was isolated from sponge samples, and seven novel 18-peptide compounds trichorzins A−G (**534**−**540**) were identified from this strain. These compounds showed strong cytotoxicity against several human cancer cell lines, including human lung adenocarcinoma A549, human non-small cell lung cancer H1299, human colorectal cancer SW480, and human pancreatic cancer SW1990, with the IC_50_ values ranging from 0.46 to 4.7 μmol/L (Lin et al. 2023). From the fungus *Trichoderma* sp., seven novel 18-residue peptaibols, neoatroviridins E−K (**541**−**547**), along with six new 14-residue peptaibols, harzianins NPDG J−O (**548**−**553**) were isolated. In antimicrobial assessments, compounds **541**−**547** exhibited moderate inhibitory activities against *S. aureus* 209P, with MIC values ranging from 8−32 μg/mL. Moreover, compound **509** exhibited moderate inhibitory effect on *C. albicans* FIM709, with a MIC value of 16 μg/mL (Cheng et al. 2023).

**Figure 24.**
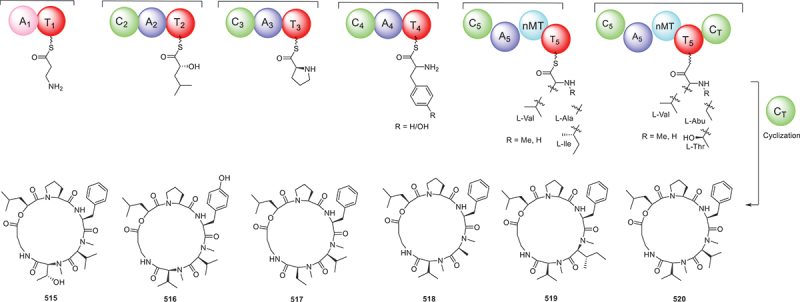
Biosynthetic pathway of compounds **515**−**520**.

According to the above analysis, it was found that compound **513** exhibited remarkable anti-inflammatory activity, while antifungal agents **516** and **517** had strong inhibitory effects on the growth of *Geotrichum citri-aurantii* mycelia. And a series of anti-tumour agents **534**−**540** significantly exhibited excellent cytotoxicity against several human cancer cell lines. Furthermore, the biosynthetic pathway of compounds **515**−**520** (Figure 24) is summarised as a case of the biosynthesis of peptides. This BGC contains a typical linear NRPS gene, which encodes six modules containing 18 domains. These modules work together to form the cyclic peptide backbone (Jiang et al. 2023c).

## 4. Discussion and conclusions

As described in this review, natural products originating from fungi exhibited a large chemical diversity and significant medicinal values. Despite encountering challenges such as limited yields, complex extracts, missing biological targets, and synthetic hurdles, fungal natural product research has made significant strides. In addition, innovative study strategies such as HPLC-MS/MS-based molecular networking, NMR-guided separations, co-culture techniques, and activation of silent BGCs (OSMAC strategy and heterologous expression) have enabled systematic exploration of fungal natural products. These approaches have led to the isolation of new compounds with diverse biological activities. The combination of these new approaches could accelerate the discovery of fungal natural products. For instance, chemical biology can be used to exploit the complex chemical architecture of natural products for the exploration of novel targets. Moreover, detailed biological investigations can facilitate the identification and subsequent screening of active natural compounds possessing diverse chemical structures (Luo et al. 2024).

Furthermore, from the perspective of biological activities, researches have mainly focused on bioactivities including cytotoxic, anti-inflammatory, anti-bacterial, anti-fungal, anti-viral, anti-parasitic, and antioxidant as well as other effects. As a result, only less than 40% of the reviewed compounds exhibited pharmacological effects. Fortunately, the combination of novel research strategies could not only identify the new action sites for active compounds, but also provide a foundation for quickly obtaining a large number of novel, low-toxicity, and efficient fungal natural products. Moreover, the origin of fungal strains is another important aspect of our concern. Among the 92 strains of fungi described in this review, approximately 37% of them remained unidentified at species levels, indicating the vast diversity of the fungal species and their enormous potential for exploration. Interestingly, more than half of the endophytic fungi were isolated from medicinal plants, which emphasises that the pursuit of excellent pharmacological activity is the ultimate objective and core significance of natural product research.

In this review, we provide an overview of the new natural products derived from fungi reported in the given journals during 2023. A total of 553 novel natural products, including 219 polyketides, 145 terpenoids, 35 steroids, 106 alkaloids, and 48 peptides are characterised by their respective chemical structures and relevant biological activities. With the rapid advancements in multi-omics techniques and artificial intelligence, researchers should combine the different techniques and study strategies to the discovery of candidate compounds from fungi. Additionally, multidisciplinary collaborations such as microbiology, synthetic biology, chemistry, pharmacology, and bioinformatics will expedite the discovery of fungal natural products with therapeutic potential.
